# New Insights into Acylhydrazones *E*/*Z* Isomerization: An Experimental and Theoretical Approach †

**DOI:** 10.3390/ijms241914739

**Published:** 2023-09-29

**Authors:** Sara Fernández-Palacios, Esther Matamoros, Isabel Morato Rojas, Juan T. López Navarrete, M. Carmen Ruiz Delgado, Yolanda Vida, Ezequiel Perez-Inestrosa

**Affiliations:** 1 Departamento de Química Física, Universidad de Málaga, Campus Teatinos s/n, 29071 Málaga, Spain; sarafpc@uma.es (S.F.-P.); teodomiro@uma.es (J.T.L.N.); 2 Departamento de Química Orgánica, Universidad de Málaga, Campus Teatinos s/n, 29071 Málaga, Spain; esthermc@uma.es (E.M.); moratorojasisabel@gmail.com (I.M.R.); inestrosa@uma.es (E.P.-I.); 3 Instituto de Investigación Biomédica de Málaga y Plataforma en Nanomedicina—IBIMA, Plataforma Bionand, Parque Tecnológico de Andalucía, 29590 Málaga, Spain

**Keywords:** acylhydrazones, *E*/*Z* isomerization, UV–Vis, NMR, Raman, DFT calculations

## Abstract

A family of acylhydrazones have been prepared and characterized with the aim of investigating their potential as information storage systems. Their well-established synthetic methodologies allowed for the preparation of seven chemically stable acylhydrazones in excellent yields that have been photophysically and photochemically characterized. In addition, DFT and TD-DFT calculations have been performed to gain more insights into the structural, energetic and photophysical properties of the *E*/*Z* isomers. Our results reveal that *E*/*Z* configurational isomerization upon irradiation is highly dependent on the stabilization of the *E* or *Z* isomers due to the formation of intramolecular H bonds and the electronic/steric effects intrinsically related to their structures. In addition, Raman spectroscopy is also used to confirm the molecular structural changes after the formation of hydrogen bonds in the isomers.

## 1. Introduction

The chemical properties and biological activity of hydrazones have attracted much attention from chemists in recent years. Hydrazones containing an azometine –C=N–NH– moiety are not only intermediates in the synthesis of various heterocyclic compounds; they are also effective organic compounds in their own right because of their biological activities and chemical and industrial versatility [1,2,3,4,5,6]. Hydrazones are easily synthetized, highly stable and possess unique chemical and structural properties due to their –C=N–NH– group, with a nucleophilic imine nitrogen, a imine carbon with both electrophilic and nucleophilic character and the possibility of configurational isomerism due to the carbon–nitrogen double bond [7]. Of particular interest are acylhydrazones, Schiff bases containing the –C=N–NH–CO– moiety, that possess a wide range of biological properties [8]. In addition, these compounds have been found to be an important class of organic materials used as hole-transporting materials [4] or sensors [9]. It has also been shown that acylhydrazones form stable chelate complexes with transition metal cations, using their oxygen and imine nitrogen as donor atoms [10]. Accordingly, additional donor sites (i.e., methoxy groups) can be introduced into their structure to increase the number of coordination sites of the resulting ligand and thus their affinity for metal ions [11,12].

Photochemical cis–trans isomerization of C=C, N=N and C=N double bonds have been investigated thoroughly [13,14]. Compounds containing the C=N group, such as imines and their derivatives, can undergo *syn–anti* isomerization, both photochemically and thermally [15,16]. In the case of hydrazones, the presence of an additional nitrogen atom that diminishes the double-bond character of the system favors *E*/*Z* isomerization [17]. However, the (*Z*)-isomer is often very unstable and is not observed, which precludes its application as a photoswitch [7,18].

An additional stabilization in the (*Z*)-isomer—for example, by introducing an intramolecular hydrogen bonding interaction—promotes a controllable isomerization [19,20]. Moreover, proper stabilization of the (*Z*)-isomer can lead to isomerization reactions controlled by pH changes or even to (*Z*)-isomers with an extremely long half-life [21,22].

On the other hand, it is also known that acylhydrazones show dynamic responses to various types of physical and chemical stimuli, showing a more feasible photochemical isomerization around the C=N double bond than hydrazones [23]. The ability of acylhydrazones to undergo constitutional, conformational and configurational dynamics makes them excellent candidates for dynamic constitutional chemistry applications [24,25]. Nevertheless, a high *E*/*Z* isomerization is only achieved when the thermodynamically less-favored (*Z*)-isomer is stabilized (i.e., via H-bond formation) or the thermodynamically favored (*E*)-isomer is destabilized [9,16,18,26]. In this regard, density functional theory (DFT) calculations have proven to be very useful for the investigation of acylhydrazones, providing a good understanding of their different conformational structures and relative stabilities [27,28,29]. On the other hand, the formation of metal complexes of related acylhydrazones with Mn(II), Fe(II), Zn(II), Co(II), Ni(II), Cu(II) and Ru(II) through the tridentate coordination site NNO has been previously described [11,30,31,32,33]. This feature, together with the C=N isomerization capabilities, allows acylhydrazones to exhibit coordination dynamics via constitutional changes of the coordination sites in the chemical structure of the *E/Z* isomers. This leads to locking and unlocking, controlled by metal ions, and therefore to the reversible transformation of different states [20].

**Figure 1:**
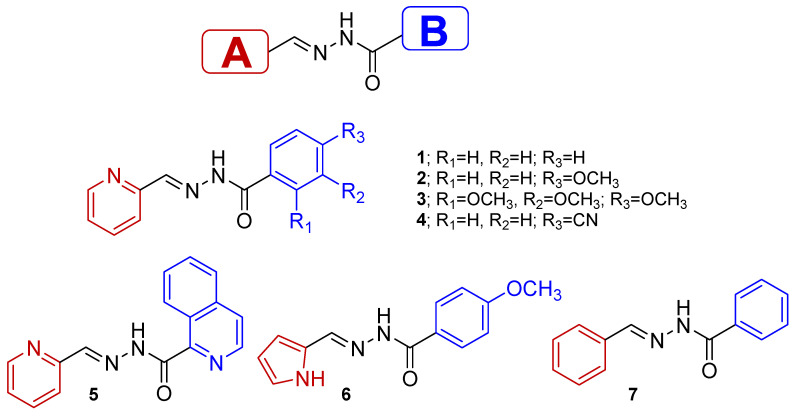
Chemical structures of the acylhydrazones **1**–**7** under study.

Acylhydrazones combine all the interesting properties described above with effective synthesis, chemical stability and a modular approach to their design. All these factors brought our attention to the study of acylhydrazones. In this work, we report the synthesis, photophysical properties and photochemical behavior of a series of substituted acylhydrazones with two ring systems (denoted as A- and B-ring systems) (see Figure 1). Different aromatic A rings have been inserted in the structure (pyridyl in **1**–**5***,* pyrrolyl in **6** and benzyl in **7**) with different abilities to stabilize the (*Z*)-isomer. Benzyl groups are used as B rings in all cases, except for compound **5**, where quinoline groups are inserted instead. Different substituents have been included in the benzoyl B ring in order to evaluate the influence of the following factors on the photochemical properties of **1**–**7** molecules: (i) the presence of acceptor (i.e., CN groups) or donor units (i.e., methoxy groups); and (ii) the stabilization of the (*E*)-isomer via the formation of the intramolecular H-bond. Furthermore, we demonstrate how the *E* and *Z* configurational states of these molecules, which may undergo photochemical interconversion, can be blocked via interactions between a metal cation and the acylhydrazones. In summary, we describe how acylhydrazones **1**–**7** are affected by physical (light) and chemical (metal cations) stimuli using a combined experimental and theoretical approach, linking various spectroscopic techniques, such as FT-Raman, UV–Vis and NMR, with DFT and time-dependent (TD-DFT) calculations.

## 2. Results and Discussion

**Figure 2:**
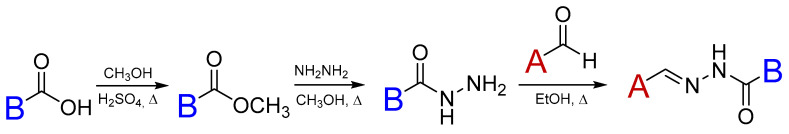
General synthesis of the acylhydrazones: **1** (B=phenyl; A=2-pyridine); **2** (B=4-methoxyphenyl; A=2-pyridine); **3** (B=2,3,4-trimethoxyphenyl; A=2-pyridine); **4** (B=4-cyanophenyl; A=2-pyridine); **5** (B=1-isoquinoline; A=2-pyridine); **6** (B=4-methoxyphenyl; A=2-pyrrolidine); **7** (R=phenyl; A=phenyl).

The synthesis of acylhydrazones is based on condensation reactions between the corresponding hydrazide and aldehyde, after which the desired compounds were isolated and purified in good yields [20,34,35]. The corresponding hydrazides were prepared using adequate carboxylic acids as starting materials, which were reacted with hydrazine hydrate after esterification with methanol (Figure 2 and Figure S1). The condensation between the obtained hydrazides with pyridine-2-carboxaldehyde (for **1**, **2**, **3**, **4** and **5**), 1H-pyrrole-2-carboxaldehyde (for **6**) or benzaldehyde (for **7**) give the desired compound as (*E*)-isomers.

**Figure 3:**
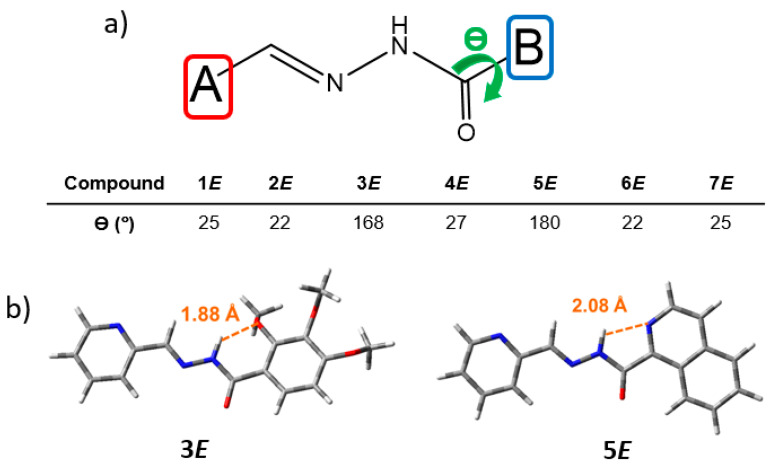
(**a**) DFT-calculated (PCM-CAM-B3LYP/6-31G** using acetonitrile as solvent) dihedral angles (°) between the B-ring and the hydrazide group of **1**–**7** (*E*)-isomers; and (**b**) top views of optimized geometries of **3** and **5** (*E*)-isomers, with the intramolecular H-bond distances shown in orange.

The DFT-optimized geometries of the configurational (*E*)-isomers of **1**–**7** compounds in acetonitrile solution were obtained at both CAM-B3LYP and B3LYP levels. Similar trends in the molecular structural description and molecular orbital energies and topologies were obtained with the two functionals; thus, the results obtained at the CAM-B3LYP are shown in the main text, while those obtained at the B3LYP level are displayed in the Supporting Information. We have tested the two dispositions (i.e., *anti*- and *syn*-) of the amide groups, with the *anti*-amide configurations found to be slightly more stable than the *syn* ones, probably due to less steric repulsion (Figure S46 and Tables S1 and S2). Then, unless otherwise indicated, all the calculations shown here in the text refer to (*E*)-isomers with *anti*-amide configurations. In all systems, the aromatic A ring is almost coplanar with the C=N double-bond planes, while the B ring is twisted with respect to the hydrazide group, except for **5*E*** (see Figure 3). The intramolecular H-bond formed between the quinoline nitrogen and the adjacent amide N-H proton (with a distance of 2.08 Å) is responsible for the perfectly coplanar conformation obtained for **5*E*** (Figure 3b). Interestingly, the formation of an H-bond between the N-H group and the methoxy group in ortho (R1 in Figure 1) is also found in **3*E*** (Figure 3b).

**Figure 4:**
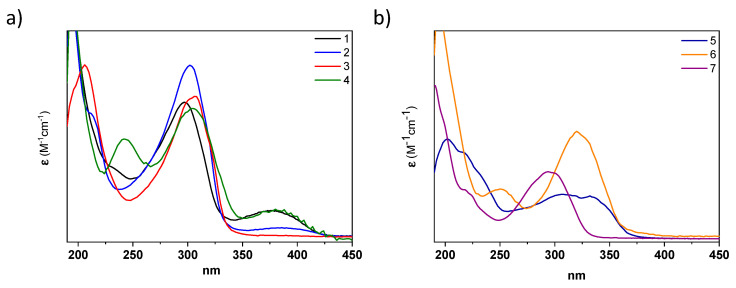
UV spectra of (**a**) **1** (black), **2** (blue), **3** (red) and **4** (green); and (**b**) **5** (dark blue), **6** (orange) and **7** (purple) *(E)*-isomers in CH_3_CN at a concentration of 5 × 10^−6^ M.

The photophysical properties of all acylhydrazones were analyzed in different solvents. The UV–Vis spectra of **1**–**4**, recorded in acetonitrile, exhibit an intense absorption band in the UV region at ~290–330 nm, with absorption coefficients of ε ~ 10^4^ M^−1^cm^−1^ typical for (hetero)aromatic highly conjugated systems (Figure 4 and Table 1). By comparing molecules **1**–**4**, the inclusion of donor (methoxy) or acceptor (CN) groups in the phenyl A ring slightly affects the absorption maxima, producing a small redshift (i.e., 6 and 4 nm when comparing **1** with **3** and **4**, respectively (see Table 1)). However, changing the type of the aromatic ring produces more significant changes. For instance, the replacement in the A ring of pyridyl in **1** by pyrrolidine in **6** produces a 20 nm bathochromic shift, while the modification of the ring B from a phenyl group in **1** to a quinoline in **5** results in a 30 nm bathochromic shift (Figure 4 and Table 1). A similar behavior was observed in methanol solutions (Figure S16 and Table 1), with a slight effect via the A-ring substituents and a moderate bathochromic shift when the B ring was modified. As expected, no significant fluorescence emission was observed in either case [36].

**Figure 5:**
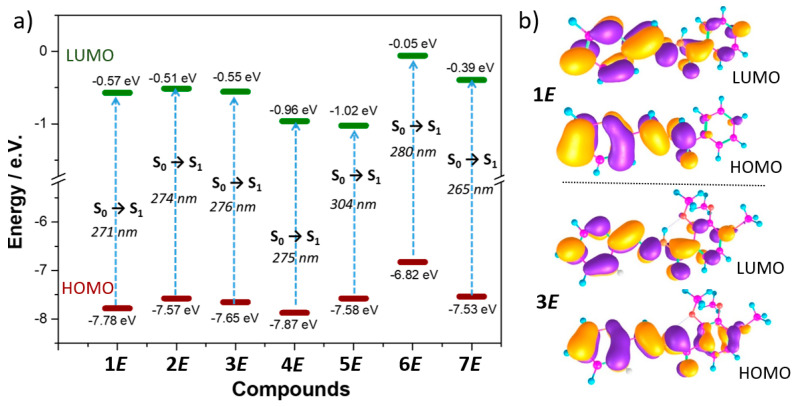
(**a**) DFT-calculated (PCM-CAM-B3LYP/6-31G** using acetonitrile as a solvent) HOMO and LUMO energies of **1**-**7** *(E)*-isomers. TD-DFT-calculated electronic absorption wavelengths (nm) for the lowest-energy electronic transitions are also shown. (**b**) The HOMO and LUMO topologies of **1*E*** and **3*E*** (for the rest of compounds, see Figure S48).

For all **1**–**7** *(E)*-isomers, TD-DFT calculations predict that the lowest-energy band (~290–330 nm) is associated to the S_0_→S_1_ electronic transition, which is assigned to a HOMO-LUMO one-electron excitation (Figure 5a). As seen in Figure 5b, the HOMOs are delocalized on the B ring and on the hydrazide group, except for **2*E***–**3*E*** and **5*E***, where they also extend on the A ring due to the insertion of methoxy donor groups on the phenyl in the former case or substitution of the phenyl by quinoline in the latter. On the other hand, the LUMOs are delocalized over the whole π-conjugated backbone (A ring + hydrazide + B ring) in all systems. Therefore, the transition towards the S_1_ state display a π–π * character.

Interestingly, TD-DFT calculations nicely reproduce the evolution of the experimental lowest-energy absorption bands with the different substitution pattern (Figure 5a). For instance, a slight bathochromic shift was found upon the insertion of methoxy or CN groups in the A ring (i.e., 5 and 4 nm when compared **1*E*** with **3*E*** and **4*E***, respectively), while a moderate bathochromic shift was predicted by changing the aromatic A ring from pyridyl to pyrrolidine or the B ring from phenyl to quinoline (i.e., 9 and 23 nm when compared **1*E*** with **6*E*** and **5*E***, respectively).

Coordination abilities of those compounds towards metal cations are expected through a tridentate NNO binding site [20]; indeed, acylhydrazone **1*E*** has been reported to form supramolecular assemblies in the presence of potassium thiocyanate [35]. Note that in **1**–**5** *(E)*-systems, three coordination sites are properly located (namely, NNO: two sp^2^-nitrogen and a sp^2^-oxigen); thus, the planar ligand can coordinate a metal cation, forming two five-membered chelate rings. In the case of **6** and **7** (*E*)-isomers, one of the sp^2^-nitrogen is lost, and only two coordination sites remain: the imine-*N* and the amide-*O* atoms.

The coordination properties of compounds **1**–**7** (*E*)-isomers with alkaline earth cations have been evaluated in acetonitrile and methanol solutions. The absorption spectra of all acylhydrazones were slightly affected by the presence of magnesium perchlorate, showing a bathochromic shift, while other cations did not produce significant variations (Figures S17–S30). In the emission profile, for acylhydrazones **1**–**4** and **7** (*E*)-isomers, the presence of Mg^2+^ induced the appearance of an extremely weak emission band with maxima at 410 nm for **1*E***, **3*E***, **4*E*** and **7*E*** or 491 nm for **2*E***, but with depreciable quantum yields. The insertion of electron donor groups (–OCH_3_) into the B ring (Figure 1) would be expected to increase the electron density in the carbonyl groups, thus increasing the metal cation binding ability of the corresponding acylhydrazones [24]; however, no substantial changes in the absorption or emission spectra have been observed.

Acylhydrazones differ from other classes of compounds containing the C=N double bond by the presence of the carbonyl group. Although one of the main photochemical reactions in carbonyl-containing aromatic compounds is photoreduction, previous work has shown that the photo- and thermal transformation of compound **7** are independent of the presence or absence of oxygen and the polarity of the solvent used, suggesting that the main photochemical process was the *E*/*Z* isomerization of the C=N group [36]. The configurational *E*/*Z* isomerization may take place by means of two processes, namely, photochemically or thermally. It has been shown that irradiation leads to conversion of the most thermodynamically stable *E* form to the less stable *Z* isomer [13,37,38,39], and that the direction and quantum yield of the reaction, as well as the generation of a given photostationary state, depends on the nature of the substituents [16,40].

**Figure 6:**
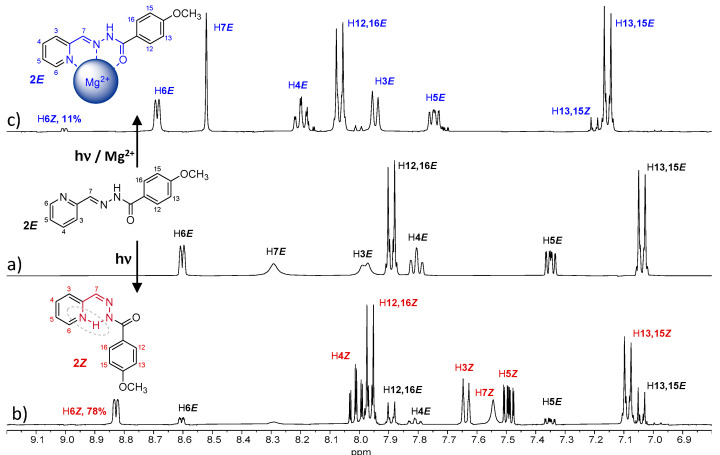
^1^H NMR spectra (400 MHz) in CD_3_CN of a solution of (**a**) ***2E*** and (**b**) **2*E*** after 45 min of UV irradiation, and (**c**) **2*E*** + 10 eq. of MgClO_4_ after 45 min of UV irradiation. The initial concentration of ***2E*** was 13 mM.

The isomerization process of **2*E*** has been evaluated in acetonitrile. The UV spectra of a 10^−5^ M solution showed important changes after 5 min of UV irradiation (Figure S31), indicating the formation of **2*Z***. Analyzing the ^1^H NMR spectra, we observed the formation of 24% of **2*Z*** after 5 min of UV irradiation, which increased to 78% after 45 min (Figure 6b and Figure S32 and Table 2); however, a reconversion to **2*E*** was observed after longer irradiation times (Supplementary Materials Figure S32). This back-isomerization from **2*Z*** to **2*E*** is probably due to the nature of the light used [23]. In this work, all irradiations were carried out using a 150W medium pressure mercury lamp without filter. Since both absorptions (**2*E*** and **2*Z***) fall within the wavelength range of the used lamp, both isomerization processes were accelerated by irradiation. Therefore, an equilibrium state between the two isomers was reached, and neither the 100% of **2*Z*** isomer was obtained nor was the complete reconversion to **2*E*** possible, since longer irradiation times did not modify the observed *E/Z* isomers ratio.

The presence of Mg^+2^ in the media significantly affected the ^1^H-NMR of **2*E***, indicating the formation of a coordination complex (Figure 6c). The most-affected signals were those corresponding to the pyridine ring (A ring in Figure 1), which is consistent with the 180º rotation that this ring undergoes when cation coordination occurs. Then, the ligand adopts a planar conformation, allowing coordination of the cation via the *N*-pyridine, *N*-imine and the *O*-amide atoms. The most significant displacements correspond to the so called H4*E,* H5*E* and H6*E*, which are displaced at low field (marked in blue in Figure 6c). The isomerization process was also affected as no conversions higher than 11% were observed when similar experiments were performed in the presence of Mg^+2^ (Figure 6 and Table 2), even with longer irradiations times, demonstrating cation-controlled locking.

As it is well-established, photochemical isomerization depends strongly on solvent polarity and its capacity to stabilize the photoinduced transition state [16]. When a solution of ***2E*** in methanol has been placed under UV light, a complete *E*-to-*Z* isomerization is observable after 45 min (Figure S33). Contrary to what was observed in acetonitrile solutions, irradiation of the sample for a longer time did not result in reconversion to ***2E*** or the formation of new products. Furthermore, no side products were observed after irradiating for more than one week, and 100 % of **2*Z*** was obtained even under preparative conditions. This indicates a high stabilization of the (*Z*)-isomer in methanol.

The presence of Mg^+2^ in the medium affects the isomerization by slowing down the conversion; however, this blocking effect is less pronounced in methanol than in acetonitrile solution. Note that the formation of the coordination complex is less favored in a protonic polar solvent, where, in addition, no appreciable differences were observed in the ^1^H-NMR spectra of a methanolic solutions of **2*E*** after the addition of MgClO_4_ (Figure S34). Therefore, the use of acetonitrile as a solvent favored the formation of the coordination complex with Mg^+2^, blocking the isomerization process more effectively, although the isomerization process in the absence of a metal cation was less effective, not reaching 100% conversions to the (*Z*)-isomer (Table 2).

At this point, we decided to use methanolic solutions to evaluate the isomerization of all acylhydrazones, since the conversions can reach 100%. As seen in Table 3, conversion to the *Z* configuration was evaluated via 120 min of UV irradiation to the *E* form.

**Figure 7:**
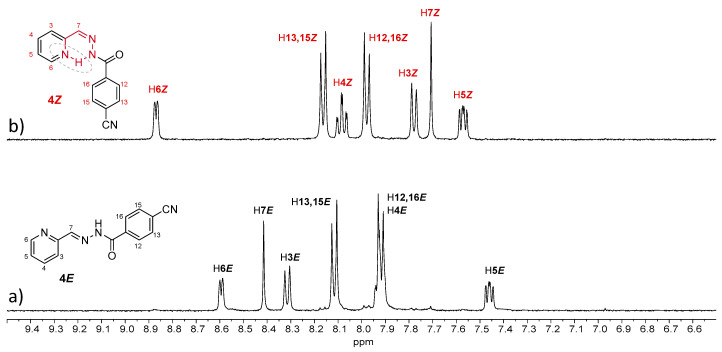
^1^H NMR spectra (400 MHz) in CD_3_OD of a solution of (**a**) **4*E*** and (**b**) **4*E*** after 45 min of UV irradiation. The initial concentration of **4*E*** was 13 mM.

Compound **4*E*** showed similar behavior to **2*E***, as irradiation of a 13 mM methanol solution under UV light induced a complete *E*-to-*Z* isomerization (Figure 7 and Table 3). However, this was not observed for the other compounds, even with longer irradiation times. For **1*E*** and **3*E***, approximately 80% of the (*Z*)-isomer was observed, and no further conversion was obtained with time (Figures S35 and S37 and Table 3). Nevertheless, both compounds behave differently, since for **3**, a *Z*-to-*E* reconversion was observed after longer irradiation times. On the other hand, the presence of Mg^+2^ in the medium affected the isomerization of **1**, **2** and **3**, slowing the conversion, with this blocking effect being more pronounced for **2** (Figures S34, S36, S38 and S39 and Table 3).

As confirmed via DFT-optimized (*Z*)-isomers of compounds **1**–**4**, the presence of the pyridine A ring allows for the formation of a six-membered intramolecular H-bond between the 2-piridyne and the amide N-H of the hydrazide moiety after isomerization (Figures S53 and S54), increasing the thermodynamic stability of the (*Z*)-isomer. Furthermore, substitution at the *para* position of the aromatic ring (B ring in Figure 1) enhances the C=N isomerization process, with 100% of (*Z*)-isomer observed for **2** and **4** (instead of 80% in the case of no substitution).

**Figure 8:**
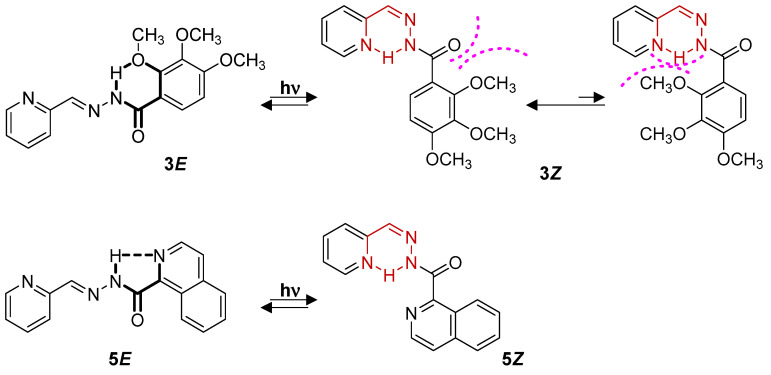
*E*/*Z* isomerization process for acylhydrazones **3** and **5**.

However, a different behavior was observed for compound **3*E***, where lower isomerization yields were obtained. This is due to the formation of an intramolecular H-bond between the N-H group and the methoxy group in the *ortho* position in the (*E*)-isomer (Figure 3 and Figure 8). This stabilization of the (*E*)-isomer makes conversion to the (*Z*)-isomer somewhat more difficult as the process requires additional activation energy, as well as promoting a partial reconversion to the (*E*)-isomer at longer irradiation times. Nevertheless, an equilibrium state between the two isomers was reached, similar to that observed for **2** in acetonitrile solution, since longer irradiation times did not modify the observed *E/Z* isomers ratio.

The electronic nature of the substituent also plays an important role. While both the electron donor (–OCH_3_) and acceptor (–CN) *p*-substituent promotes a complete *E*/*Z* interconversion in **2** and **4**, the behavior of both compounds in the presence of Mg^+2^ differs considerably. While the blocking of the *E* configuration was important for **2** (donor *p*-substituent), with a decrease in the percentage of (*Z*)-isomer reached, in the case of **4** (*p*-substituent acceptor), a complete conversion was always observed. This indicates the formation of a more effective coordination complex in the case of **2**. However, for **1** and **3** compounds, no considerable effects are observed in the presence of Mg^+2^, indicating that the formation of the complex was not successful for those compounds.

For compound **5**, the *E/Z* conversion was lower than that of the previous compounds (Figure S40 and Table 3). In this case, the quinoline ring promotes the formation of an intramolecular H-bond in the (*E*)-isomer (Figure 3 and Figure 8), and as in the case of **3**, the conversion to the (*Z*)-isomer is less favored [36]. However, both compounds evolve differently after irradiation, with **5** showing a slower isomerization, with conversions of 66% only after long irradiation times. Furthermore, no reconversion to the (*E*)-isomer was observed. This could indicate a higher stabilization for **5*Z*** than for **3*Z***, probably due to steric hindrance in **3*Z*** due to the bulky *ortho* substitutes. Surprisingly, the presence of Mg^+2^ improved the yield, with a conversion of 55% instead of 35% in the absence of the cation (Figure S41 and Table 3). This seems to indicate that the presence of the cation breaks up or hinders the formation of the intramolecular H-bond in **5*E***.

**Figure 9:**
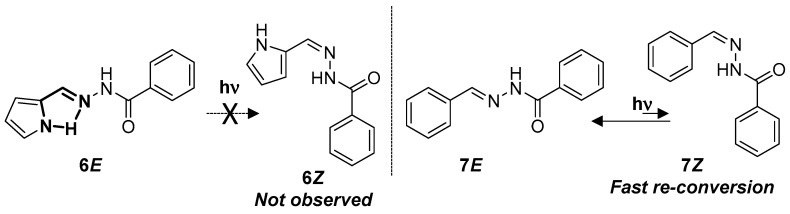
*E*/*Z* isomerization process for acylhydrazones **6** and **7**.

For compounds **6** and **7**, which differ from the rest in the A ring (Figure 1), no isomerization was observed (Figures S42–S45 and Table 3). In both cases, the absence of a stabilization in the (*Z*)-isomer has a strong influence in the process (Figures S55 and S56). However, in the case of compound **6**, the pyrrolyl group promotes the stabilization of **6*E*** via the formation of an intramolecular H-bond, which, together with the lack of stabilization of **6Z**, makes the isomerization process almost negligible (Figure 9). In addition, longer irradiation times lead to the formation of undesirable side-products instead of the (*Z*)-isomer (Figure S42). In the case of **7**, no isomerization or side product formation was observed, even at longer irradiation times (Figure S44). However, it has been previously reported that irradiations at 297 nm of **7*E*** give rise to **7*Z***, and irradiations to the thermodynamically less stable **7*Z*** at 365 nm regenerate **7*E***. In fact, **7*Z*** is thermally unstable [23]. In our case, the nature of the light used in our experiments promotes isomerization in both directions, and the life time of **7*Z*** was not long enough to be detected under the conditions used.

**Figure 10:**
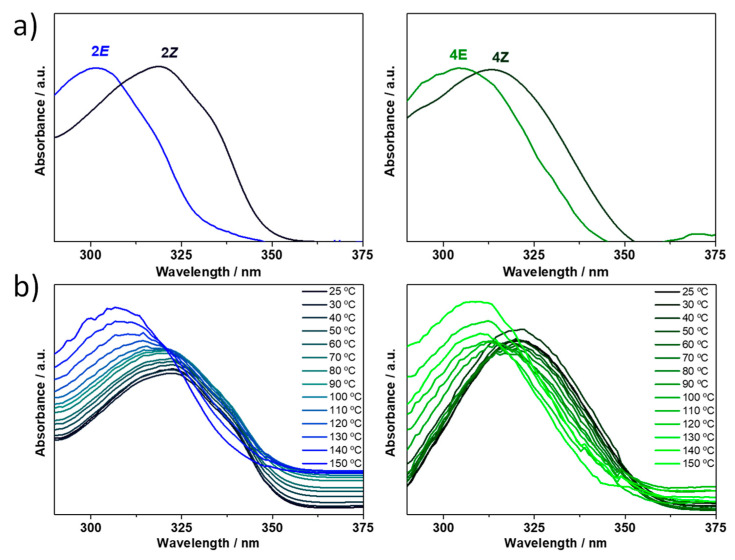
(**a**) UV–Vis absorption spectra of the photoisomerization experiment of acylhydrazones **2** (left) and **4** (right) in CHCl_3_ performed via UV irradiation with λ = 365 nm for 45 min. (**b**) UV–Vis spectral changes accompanying the thermal conversion of the (*Z*)-isomer to the (*E*)-isomer in DMSO upon heating for **2** (left) and **4** (right) compounds.

As irradiation of acylhydrazones **2** and **4** results in a complete *E*-to-*Z* photosiomerization, we now investigate in more detailed the *E*/*Z* interconversion in these two compounds. As seen in Figure 10a, the lowest energy absorption band is bathochromically shifted upon UV light irradiation, going from the *E* to the *Z* isomer (i.e., 16 nm for **2** and 9 nm in **4**). This bathochromic shifting of the (*Z*)-isomer’s absorption with respect to the more stable (*E*)-isomer leads to so-called negative photochromism [41], which has already been observed in other acylhydrazone systems [23]. This absorption maxima redshift is well reproduced by TD-DFT calculations (Figures S57 and S58) and can be ascribed to a more extended π-conjugation in the (*Z*)-isomer as a result of the formation of a six-membered H-bonded network (HN-N=C-C=N), as seen in Figure 8. Moreover, we aim to inspect the influence of temperature on the *E*→*Z* configurational isomerization. To this end, the solution of (*Z*)-isomer obtained after UV irradiation was heated up to 150 °C, generating the (*E*)-isomer (Figure 10b), and thus showing the reversibility of the *E*/*Z* interconversion in compounds **2** and **4**.

**Figure 11:**
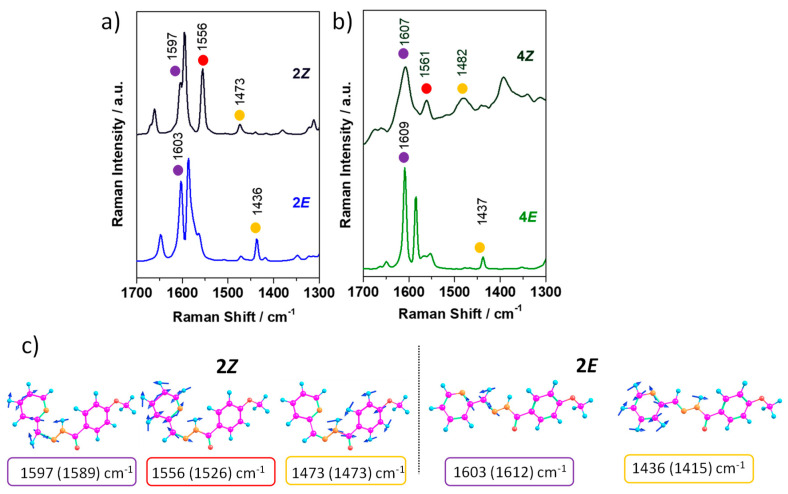
Solid-state FT-Raman ((λexc = 1064 nm) spectra for *E* and *Z* isomers of **2** (**a**) and **4** (**b**) compounds. (**c**) PCM-CAM-B3LYP/6-31G** vibrational eigenvectors associated with the Raman features discussed in the text, taking **2*E*** and **2*Z*** isomers as representative examples. The experimental and theoretical (in parentheses) wavenumbers are also shown.

Finally, we investigate the molecular structural changes associated with the *E*→*Z* interconversion using Raman spectroscopy. Bond length changes and π-electron density redistribution are expected to occur upon *E*/*Z* isomerization, which might result in changes to the Raman spectral profile (i.e., changes in peak position and relative peak intensities). Please note that despite the interesting properties that can be retrieved from the Raman analysis, there are only a few papers in the literature investigating the Raman spectroscopic properties of photochromic hydrazones or acylhydrazones [42,43]. Figure 11 compares the FT-Raman spectrum of *E* and *Z* isomers for **2** and **4**. As a result of the *E/Z* interconversion, the following changes in the Raman spectral features were observed: (i) the frequency of the C=N stretching (denoted with a purple circle) shifts towards lower values in the (*Z*)-isomers due to an increased pi-delocalization when compared to the (*E*)-isomers; (ii) a new intense band appears at around 1560 cm^−1^ in the *Z* form, marked with a red circle, which is associated with pyridine ring stretching coupled with C=N stretching and the NH bending normal modes; this is ascribed to the formation of a H-bond six-membered fragment (HN-N=C-C-N) in the (*Z*)-isomer; and iii) the bending of the NH group (denoted with a yellow circle) is shifted to higher frequencies upon *E*/*Z* interconversion due to the presence of the intramolecular H-bonding interaction, which also results in a different structural environment around this link; note that this mode is mixed with C-N and phenyl ring stretching in the case of the (*Z*)-isomer, while in the (*E*)-isomer, it is coupled with pyridine ring stretching. This vibrational assignment has been achieved thanks to the good agreement found between the theoretical and experimental Raman spectra (see Figure S59).

## 3. Conclusions

Several acylhydrazones have been prepared and characterized with structural differences that may stabilize their *E* or *Z* form. DFT calculations demonstrate the formation of intramolecular H-bonds which, in turn, would affect the stability of the isomers. Studies of *E*/*Z* configurational isomerization via UV–Vis, NMR and Raman spectroscopy show that although the stabilization of the *E* or *Z* isomers is a determining factor, the process is also strongly influenced by electronic and steric factors. While *p*-substitution in the B ring undergoes a complete isomerization to the *Z* form, the insertion of a bulky group (*o*-position substituent or a bigger-size B ring) affects the process due to the steric hindrance slowing down the conversion. In addition, *E*/*Z* isomerization is also affected by solvent effects and intermolecular interactions since, in the presence of Mg^+2^, the cation locking effect on isomerization is clear, although no major influence on the UV–Vis spectra has been observed. In summary, acylhydrazones are very interesting compounds capable of combining efficient synthesis, chemical stability and a modular approach in their design, with extraordinary properties such as the ability to undergo constitutional, conformational or configurational dynamics, thus constituting an excellent scaffold for the design of more complex functional materials. The exhaustive experimental and computational study carried out in this work could be of great help in better understanding the *E*/*Z* isomerization process in acylhydrazones, with the aim of identifying new molecular design strategies of acylhydrazone-based switches with potential use in different fields of science.

## 4. Materials and Methods

### 4.1. General Consideration

All reagents and solvents were used without further purification (except 2-pyridinecarbaldehyde via distillation). Preparative thin-layer chromatography was made using silica plates (60 F254) and revealed with UV light (254 nm). Melting points were recorded on a Gallenkamp apparatus. ^1^H NMR spectra were performed in a Bruker WP-200 SY at 200 MHz for ^1^H and 50.3 MHz for ^13^C or in a Bruker Biospin Avance III 400 at 400 MHz for ^1^H and 100.6 MHz for ^13^C, with the solvent peak used as the internal reference. Mass spectra were recorded in a mass spectrometer with electronic impact ionization (70 eV) Thermo Scientific DSQ II single Quadrupole GC/MS with focus GC. Absorption spectra were measured with an HP 8452 Diode Array Spectrophotometer. Emission spectra were measured with a Jasco FP-750 Spectrophotometer. Fluorescence quantum yields, ɸ_F_, were measured for all the solutions using as reference quinine sulphate in 0.1 M H_2_SO_4_ (ɸ_F_ = 0.55). Irradiations were carried out using a water-cooled 150W medium-pressure mercury lamp in an immersion reactor. The FT-Raman spectra for for *E* and *Z* isomers of **2** and **4** compounds were recorded using an FT-Raman accessory kit (RamII)) linked to a Bruker Vertex70 spectrometer and a Nd-YAG laser source with excitation at λ = 1064 nm, with a germanium detector operating at liquid-nitrogen temperature. The operating power for the exciting laser radiation was kept at a level lower than 100 mW in all experiments. Raman scattering radiation was collected by averaging 1000 scans with 4 cm^−1^ spectral resolution of 4 cm^−1^.

### 4.2. DFT and TD-DFT Calculations

The ground-state molecular geometries, vibrational frequencies and Raman intensities of *E* and *Z* isomers for acylhydrazones **1**–**7** were optimized within the framework of the density functional theory (DFT) using the hybrid, generalized gradient approximation (GGA) functional B3LYP [44,45] and the CAM-B3LYP [45] long-range corrected hybrid functional together with the 6-31G** basis set [46,47], as implemented in the GAUSSIAN16 program [48]. All geometrical parameters were allowed to vary independently, and no imaginary harmonic modes were observed in the spectrum, indicating that each optimized structure is a local minimum on the corresponding structural potential-energy surface. The effects of solvents were taken into account in order to reproduce more accurately the experiments carried out in solution. For this purpose, the SCRF (self-consistent-reaction-field) theory and the PCM (polarized continuum model [49]) approach were applied to simulate the interaction between the molecules and the solvent (chloroform).

The time-dependent DFT (TD-DFT) [50,51] approach was used on the resulting optimized geometries to compute the vertical electronic excitation energies. Absorption spectra were modeled by the convolution of the vertical transition energies and oscillator strengths with Gaussian functions (by using 0.3 eV width at the half-height) through the GaussSum 3.0 software [52]. Molecular orbital distributions were plotted using the ChemCraft 1.8 molecular modeling software [53].

Interestingly, both CAM-B3LYP and B3LYP functionals give similar trends in the description of the molecular structure (Figure 3 and Figure S47), the HOMO and LUMO energies and topologies (Figure 5 and Figures S48–S50) and the characterization of the vertical electronic transitions (Figures S51 and S52) for the whole family of acylhydrazones under study.

### 4.3. General Procedure for the Synthesis of Esters

Carboxylic acids (10 mmol) were dissolved in methanol (20 mL) and H_2_SO_4_ was added. The reaction mixture was heated to reflux for 24 h under N_2_ atmosphere. The solvent was removed and the obtained crude mixture was dissolved in dichloromethane and washed with NaHCO_3_ 5%. The aqueous layer was extracted with dichloromethane. The combined organic layers were dried over MgSO_4,_ and the solvent was evaporated to obtain the corresponding ester: Methyl benzoate**:** Benzoic acid (25mmol, 3g) and H_2_SO_4_ (19 mmol, 1 mL); colorless oil (3g, 88% yield). Methyl 4-methoxybenzoate: 4-methoxybenzoic acid 1 (9.8 mmol, 1.5g) and H_2_SO_4_ (7.35 mmol, 0.4 mL); white solid (1.31g, 80% yield). Methyl 2,3,4-trimethoxybenzoate: 2,3,4-trimethoxybenzoic acid 3 (7 mmol, 1.5 g) and H_2_SO_4_ (5.3 mmol, 0.3 mL); colorless oil (1.45g, 90% yield). Methyl 4-cyanobenzoate: 4-cyanobenzoic acid 5 (13 mmol, 2 g) and H_2_SO_4_ (10 mmol, 0.6 mL); yellow solid (2g, 96% yield). Methyl isoquinoline-1-carboxylate: isoquinoline-1-carboxylic acid 2 (4 mmol, 0.7 g) and H_2_SO_4_ (3 mmol, 0.2 mL); yellow solid (0.53 g, 70% yield).

### 4.4. General Procedure for the Synthesis of Hydrazides

Methyl esters (6 mmol) were dissolved in methanol (5 mL), and hydrazine hydrate was added (60 mmol). The reaction mixture was heated to reflux for 24 h. The solvent was removed, and the obtained crude mixture was dissolved in dichloromethane and washed with water. The organic layer was dried over MgSO_4_, and the solvent was removed to obtain the corresponding hydrazides [54]: Benzohydrazide: ethyl benzoate (22 mmol, 3g) and hydrazine hydrate (44 mmol, 2.1 mL); white solid (2.64 g, 88% yield). 4-Methoxybenzohydrazide: methyl 4-methoxybenzoate (16 mmol, 2.63 g) and hydrazine hydrate (32 mmol, 1 mL); white solid (1.22 g, 46% yield). 2,3,4-Trimethoxybenzohydrazide: methyl 2,3,4-trimethoxybenzoate (6mmol, 1.4 g) and hydrazine hydrate (60 mmol, 1.9 mL); a yellow solid was obtained after recrystallization in ethyl acetate (1.19 g, 85% yield). 4-Cyanobenzohydrazide: methyl 4-cyanobenzoate (0.62 mmol, 0.1 g) and hydrazine hydrate (47 mmol, 1.5 mL); a white solid was obtained after recrystallization in ethanol/water (0.074g, 75% yield). Isoquinoline-1-carbohydrazide: methyl isoquinoline-1-carboxylate (2.8 mmol, 0.53 g) and hydrazine hydrate (28 mmol, 0.8 mL); white solid (0.43 g, 82% yield).

### 4.5. General Procedure for the Synthesis of Acylhydrazones [20]

Hydrazides (1.89 mmol) were placed in a two-necked round-bottom flask under Ar atmosphere and dissolved in 10 mL of absolute ethanol. Corresponding aldehyde (1.89 mmol) was added, and the reaction mixture was stirred for 24 h at reflux. After this time, the solvent was removed, and the reaction crude was purified via recrystallization or flash column chromatography. In all cases, the *E* isomer was obtained. NMR spectra are shown in Supplementary Materials Figures S2–S15.

(*E*)-*N*’-(pyridin-2-ylmethylene)benzohydrazide (1) [20]: Benzohydrazide (7.4 mmol, 1 g) and pyridine-2-carboxaldehyde freshly distilled (7.4 mmol, 0.7 mL); **1** was obtained after recrystallization in ethyl acetate/methanol (0.84 g, 50% yield); colorless solid. M.p.: 120–121 °C. UV–Vis (CH_3_CN) λ_max_ nm (ε): 298 (28000). ^1^H-NMR (400 MHz, CD_3_OD): δ 7.46 (m, 1H), 7.55 (t, *J* = 8 Hz, 2H), 7.63 (t, *J* = 8 Hz, 1H), 7.92 (m, 1H), 7.97 (m, 2H), 8.32 (d, *J* = 8 Hz, 1H), 8.42 (s, 1H), 8.59 (m, 1H). ^13^C-NMR (100 MHz, CD_3_OD): δ 114.3, 118.3, 120.1, 124.7, 128.6, 132.7, 137.0, 137.3, 149.1, 149.6, 153.1, 162.1. MS-EI m/z (%): 226 (M+1, 2), 225 (M+, 4), 121 (11), 120 (100), 105 (70), 92 (37), 77 (43), 65 (14).

(*E*)-4-methoxy-*N*’-(pyridin-2-ylmethylene)benzohydrazide (2) [20]: 4-Methoxybenzohydrazide (1.89 mmol, 0.31 g) and pyridine-2-carboxaldehyde freshly distilled (1.89 mmol, 0.2 mL). **2** was obtained after recrystallization in ethyl acetate/methanol (0.29 g, 60% yield); colorless solid. M.p.: 130–131 °C. UV–Vis (CH_3_CN) λ_max_ nm (ε): 302 (29100). ^1^H-NMR (400 MHz, CD_3_OD): δ 3.90 (s, 3H, -OCH_3_), 7.08 (d, *J* = 9 Hz, 2H), 7.45 (m, 1H), 7.92 (t, *J* = 8 Hz, 1H), 7.97 (d, *J* = 9 Hz, 2H), 8.34 (d, *J* = 8 Hz, 1H), 8.39 (s, 1H), 8.58 (d, *J* = 8 Hz, 1H). ^13^C-NMR (100 MHz, CD_3_OD): δ 55.8, 114.6, 121.8, 125.3, 125.5, 130.6, 138.2, 148.3, 149.7, 154.2, 164.2, 166.5. MS-EI m/z (%): 255 (M+, 18), 135 (100), 120 (42), 92 (21), 77 (15).

(*E*)-2,3,4-trimethoxy-*N*’-(pyridin-2-ylmethylene)-benzohydrazide (3): 2,3,4-Trimethoxybenzohydrazide (0.44 mmol, 0.1 g) and pyridine-2-carboxaldehyde freshly distilled (0.44 mmol, 0.05 mL); **3** was obtained (0.56 g, 40% yield) after purification via column chromatography (ethyl acetate/methanol 9:0.3); colorless solid. M.p.: 160–161 °C. UV–Vis (CH_3_CN) λ_max_ nm (ε): 306 (23828). ^1^H-NMR (400 MHz, CDCl_3_): δ 3.88 (s, 3H), 3.92 (s, 3H), 4.04 (s, 3H), 6.81 (d, *J* = 9 Hz, 1H), 7.27 (m, 1H), 7.74 (m, 1H), 8.00 (d, *J* = 9 Hz, 1H), 8.21 (m, 2H), 8.59 (d, *J* = 5 Hz, 1H). ^13^C-NMR (100 MHz, CDCl3): δ 56.2, 61.1, 62.1, 108.0, 117.2, 121.4, 124.3, 127.5, 136.6, 141.7, 147.8, 149.3, 152.3, 153.2, 157.4, 161.8. MS-EI m/z (%): 316 (M+1, 2), 315 (M+, 9), 196 (11), 195 (100), 152 (11), 120 (50).

(*E*)-4-cyano-*N*’-(pyridin-2-ylmethylene)benzohydrazide (4): 4-cyanobenzohydrazide (1.86 mmol, 0.3 g) and pyridine-2-carboxaldehyde freshly distilled (1.86 mmol, 0.18 mL); **4** was obtained (0.25 g, 53% yield) after purification via column chromatography (dichloromethane/methanol 9:0.2); colorless solid. M.p.: 215–218 °C. UV–Vis (CH_3_CN) λ_max_ nm (ε): 242 (16600), 306 (21700), 380 (4590). ^1^H-NMR (400 MHz, DMSO): δ 7.45 (m, 1H), 7.91 (t, *J* = 7 Hz, 1H), 8.00 (d, *J* = 7 Hz, 1H), 8.07 (m, 4H), 8.48 (s, 1H), 8.64 (d, *J* = 7 Hz, 1H). ^13^C-NMR (100 MHz, DMSO): δ 114.7, 118.8, 120.5, 125.1, 129.1, 133.1, 137.4, 137.7, 149.5, 150.1, 153.5, 162.5. MS-EI m/z (%): 250 (M+, 2), 130 (28), 120 (100), 102 (33), 92 (52), 65 (24).

(*E*)-*N*’-(pyridin-2-ylmethylene)isoquinoline-1-carbohydrazide (5). Isoquinoline-1-carbohydrazide (1.07 mmol, 200 mg) and pyridine-2-carboxaldehyde freshly distilled (1.07 mmol, 0.1 mL); **5** was obtained after recrystallization in ethyl acetate/ethanol (0.14 g, 40% yield); yellow solid. M.p.: 184–185 °C. UV–Vis (CH_3_CN) λ_max_ nm (ε):332 (19580). ^1^H-NMR (400 MHz, CDCl_3_): δ 7.31 (dd, *J* = 7 Hz, 5 Hz; 1H), 7.77 (m, 3H), 7.90 (m, 2H), 8.30 (d, *J* = 8 Hz, 1H), 8.36 (s, 1H), 8.54 (d, *J* = 6 Hz, 1H), 8.64 (d, *J* = 5 Hz, 1H), 9.67 (m, 1H), 11.40 (s, 1H). ^13^C-NMR (100 MHz, CDCl3): δ 121.3, 124.4, 125.3, 126.9, 127.0, 127.6, 129.3, 130.7, 130.8, 136.5, 137.5, 140.1, 148.4, 149.4, 153.1. MS-EI m/z (%): 277 (M+1, 10), 276 (M+, 38), 248 (52), 220 (56), 219 (41), 157 (20), 129 (100), 128 (58), 120 (48), 92 (42).

(*E*)-4-methoxy-*N*’-(1H-pyrrol-2-ylmethylene)-benzohydrazide (6). 4-Methoxybenzohydrazide (1.2 mmol, 0.2 g) and 1H-pyrrole-2-carboxaldehyde (1.2 mmol, 0.12 g); **6** was obtained (0.22 g, 82% yield) after purification via chromatography (ethyl acetate/cyclohexane 1:1); colorless solid. M.p.: 125–126 °C. UV–Vis (CH_3_CN) λ_max_ nm (ε):320 (9820). ^1^H-NMR (400 MHz, CDCl_3_): δ 3.86 (s, 3H), 6.24 (m, 1H), 6.49 (m, 1H), 6.95 (m, 3H), 7.83 (d, *J* = 8 Hz, 2H), 8.04 (s, 1H), 9.31 (bs, 1H), 10.05 (bs, 1H). ^13^C-NMR (100 MHz, CD_3_OD): δ 54.6, 109.1, 113.6, 114.2, 122.2, 124.6, 127.5, 129.1, 139.8, 163.0, 165.2. MS-EI m/z (%): 244 (M+1, 3), 243 (M+, 24), 151 (20), 135 (100), 107 (11), 77 (12).

(*E*)-*N*’-benzylidenebenzohydrazide (7) [35]. Benzohydrazide (7.4 mmol, 1 g) and benzaldehyde freshly distilled (7.4 mmol, 0.75 mL); **7** was obtained after recrystallization in ethyl acetate/methanol (1.37 g, 83% yield); colorless solid. M.p.: 120–121 °C. UV–Vis (CH_3_CN) λ_max_ nm (ε): 300 (27500). ^1^H-NMR (400 MHz, CD_3_OD): δ 7.45 (m, 3H), 7.54 (t, *J* = 8 Hz, 2H), 7.63 (t, *J* = 8 Hz, 1H), 7.86 (m, 2H), 7.96 (d, *J* = 8 Hz, 2H), 8.37 (s, 1H). ^13^C-NMR (100 MHz, CD_3_OD): δ 129.0, 130.1, 131.9, 133.6, 134.4, 135.7, 151.0, 167.4. MS-EI m/z (%): 225 (M+1, 2), 224 (M+, 7), 121 (46), 105 (100), 77 (34).

## Figures and Tables

**Table 1:** Absorption maxima and molar absorption coefficient of the acylhydrazones in CH_3_CN and CH_3_OH.

CH_3_CN	1	2	3	4	5	6	7
λ_max_ *	300, 378	302	306	242, 304, 380	300, 330	250, 320	294
ε	2.25, 4.40	2.91	2.38	1.66, 2.18, 4.59	1.95	2.27, 4.91	3.06
CH_3_OH	**1**	**2**	**3**	**4**	**5**	**6**	**7**
λ_max_ *	305	274, 319	319, 361	319, 365	327, 370	327	298
ε	4.42	3.38, 4.04	3.44, 1.19	2.82, 2.00	2.47, 2.83	5.14	3.08

* λ _abs max_ (nm); ε (10^4^ M^−1^cm^−1^).

**Table 2:** % of **2*Z*** isomer after UV light irradiation of the corresponding **2*E*** form (13 mM acetonitrile solutions).

Irradiation Time (min)	5	15	30	45	75	120
CH_3_CN	24%	54%	73%	78%	74%	69%
CH_3_CN + [Mg^+2^] *	11%	11%	11%	11%	11%	11%

* In the presence of 10 equivalents of Mg(ClO_4_)_2_.

**Table 3:** Percentage of *Z* isomer after UV-light irradiation of the corresponding *E* form (13 mM methanol solutions).

Irradiation Time	1	2	3	4	5	6	7
45 min	80%	100%	80%	100%	35%	0%	0%
120 min	80%	100%	67%	100%	66%	--	0%
45 min + [Mg+2] *	75%	63%	80%	100%	55%	0%	0%

* In the present of 10 equivalents of Mg(ClO_4_)_2_.

## Data Availability

Data is contained within the article or Supplementary Materials.

## Supplementary Materials

The following supporting information can be downloaded at https://www.mdpi.com/1422-0067/24/19/14739/s1.
