# Supplementary material for: New Insights into Acylhydrazones E/Z Isomerization: An Experimental and Theoretical Approach
Source: Int J Mol Sci. 2023 Sep 29;24(19):14739. doi: 10.3390/ijms241914739 (PMC10648745; doi:10.3390/ijms241914739)
Supplement: Supplementary file 1 [file ijms-24-14739-s001.zip › ijms-2604402-supplementary.pdf]

Supporting Information

New Insights into Acylhydrazones *E*/*Z* isomerization: An Experimental and Theoretical Approach

Table of Contents

	Page
**1. Synthesis**	S2
**2. NMR spectra**	S3
**3. Photophysical properties**	S10
**4. Photophysical properties in presence of metallic cations**	
**4.1 In acetonitrile solutions**	S10
**4.2 In methanol solutions**	S12
**5. Photochemical properties**	S15
**6. DFT and TD-DFT calculations**	S22

1. Synthesis of acylhydrazones 1-7

The synthesis of acyl-hydrazones **1**-**7** have been made following the procedure described in figure S1.

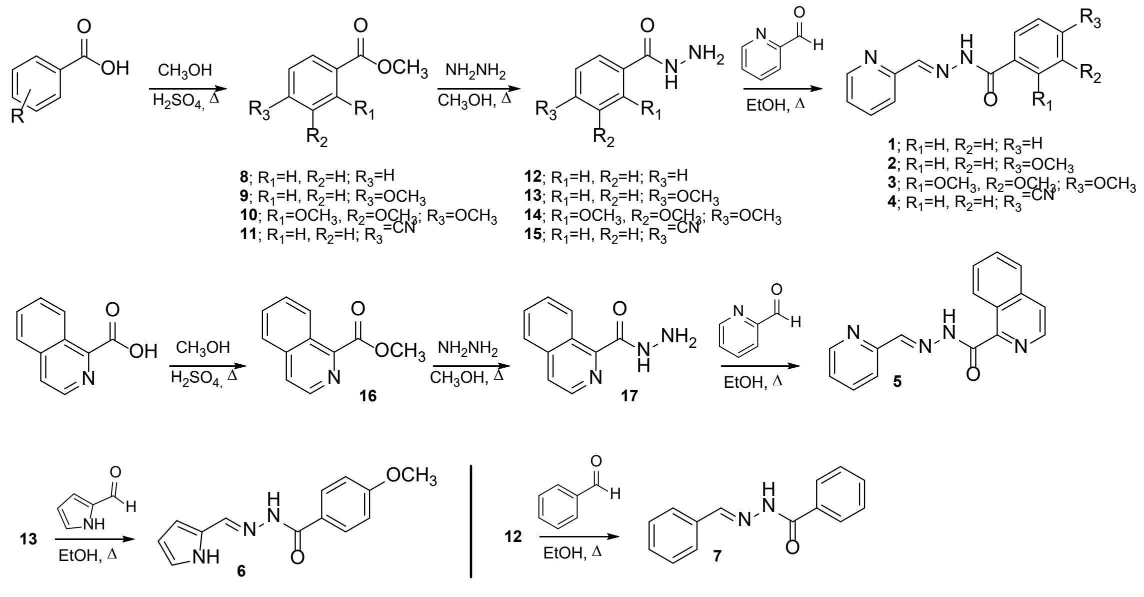

Figure S1. Synthesis of the acylhydrazones **1**-**7**.

2. NMR spectra of acylhydrazones 1-7

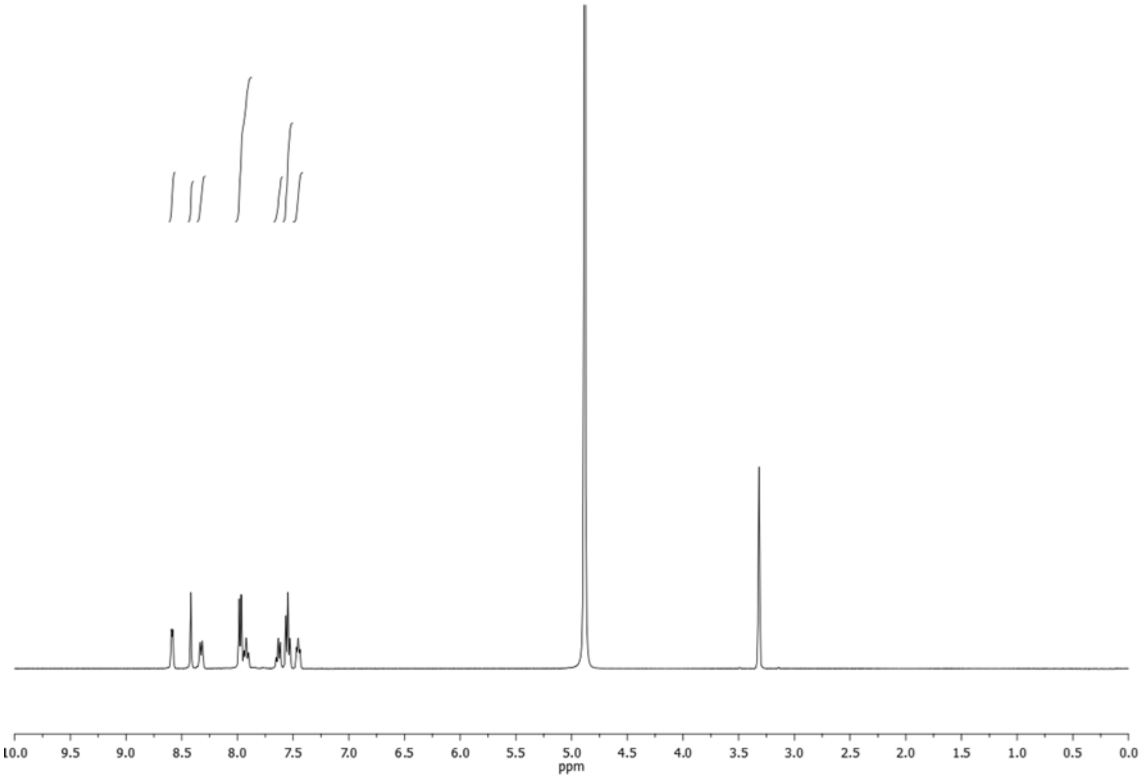

Figure S2. ^{1}H NMR spectra (400 MHz) in CD_3OD of **1*E***

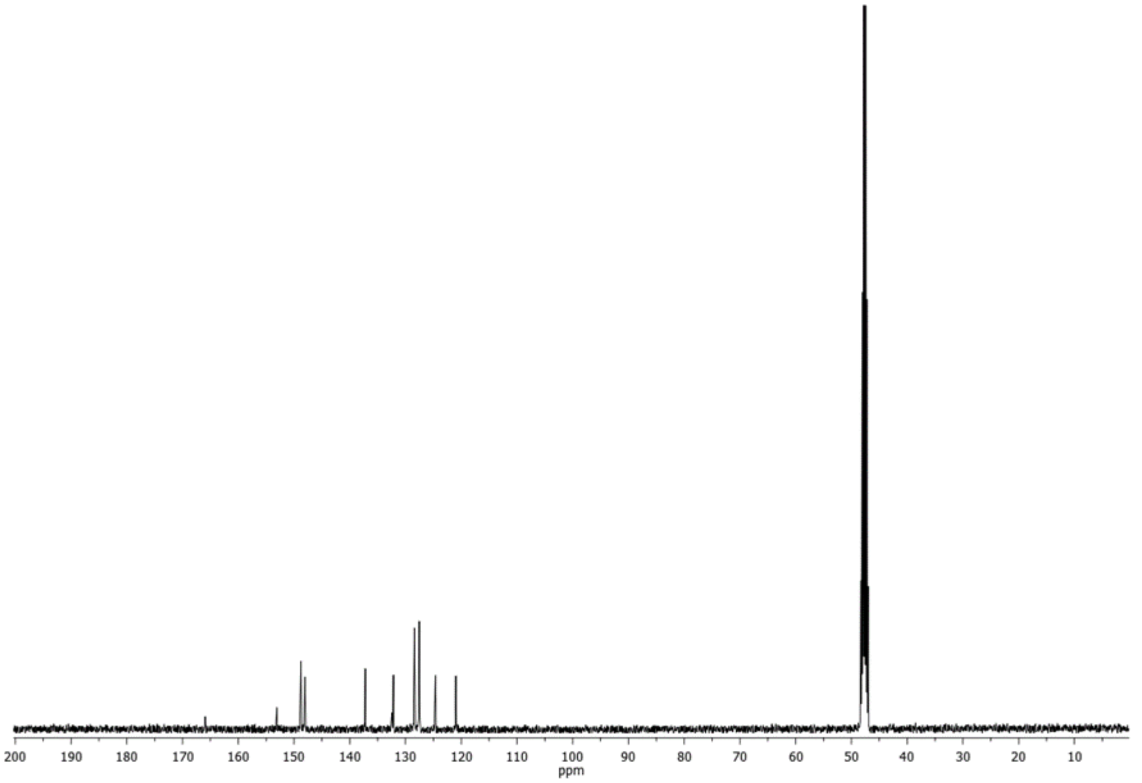

Figure S3. ^{13}C NMR spectra (100 MHz) in CD_3OD of **1*E***

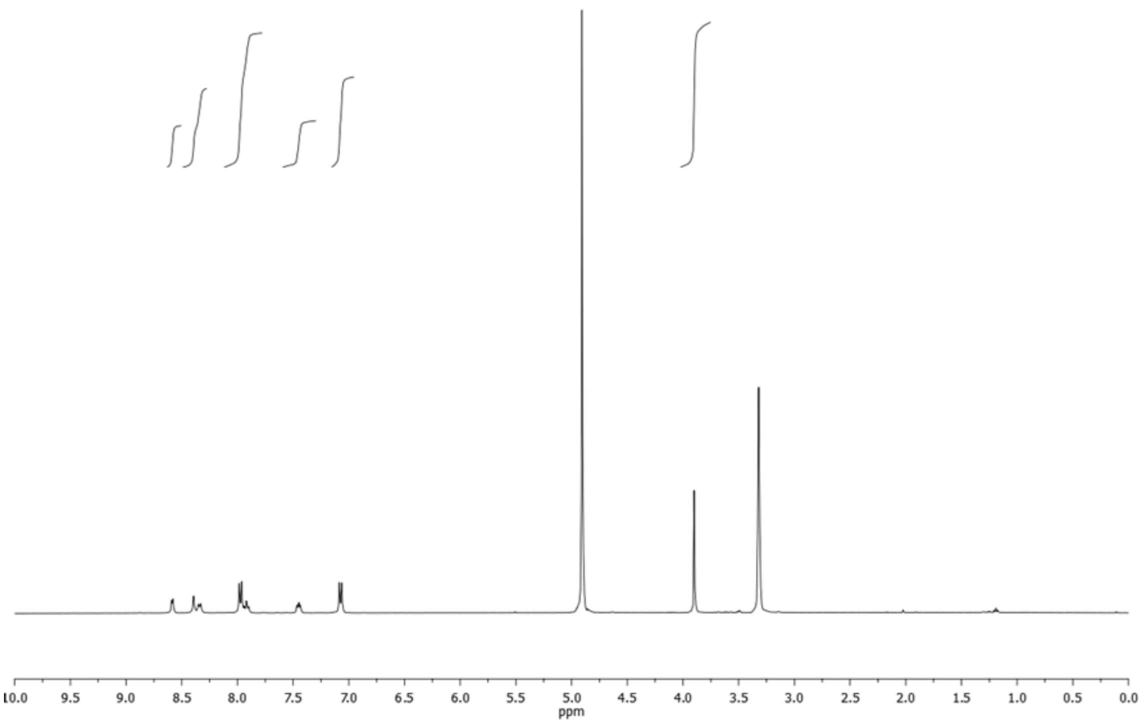

Figure S4. ^{1}H NMR spectra (400 MHz) in CD_3OD of **2*E***

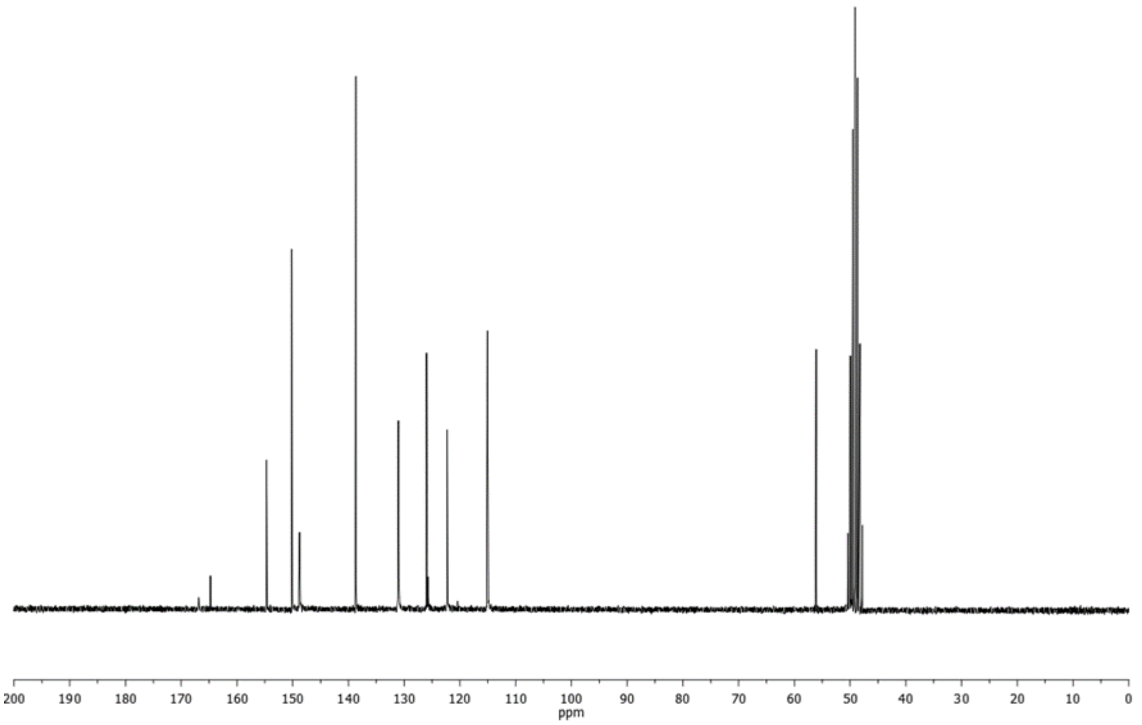

Figure S5. ^{13}C NMR spectra (100 MHz) in CD_3OD of **2*E***

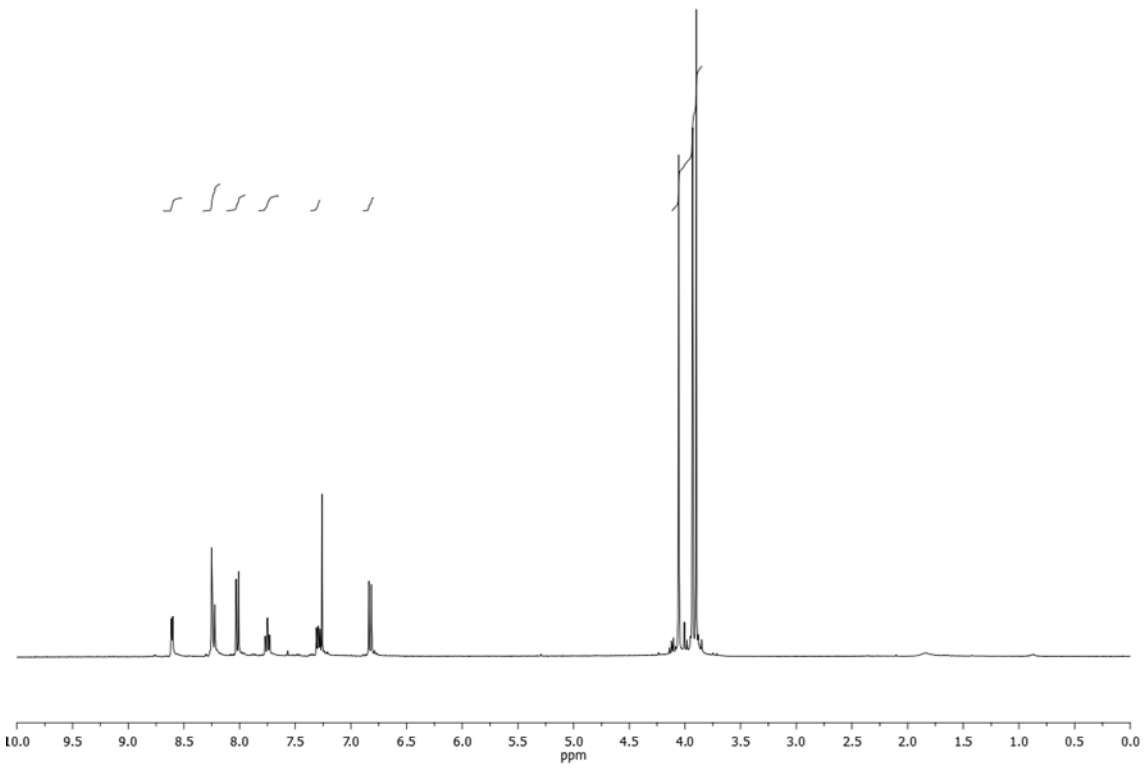

Figure S6. ^{1}H NMR spectra (400 MHz) in $CDCl_3$ of **3*E***

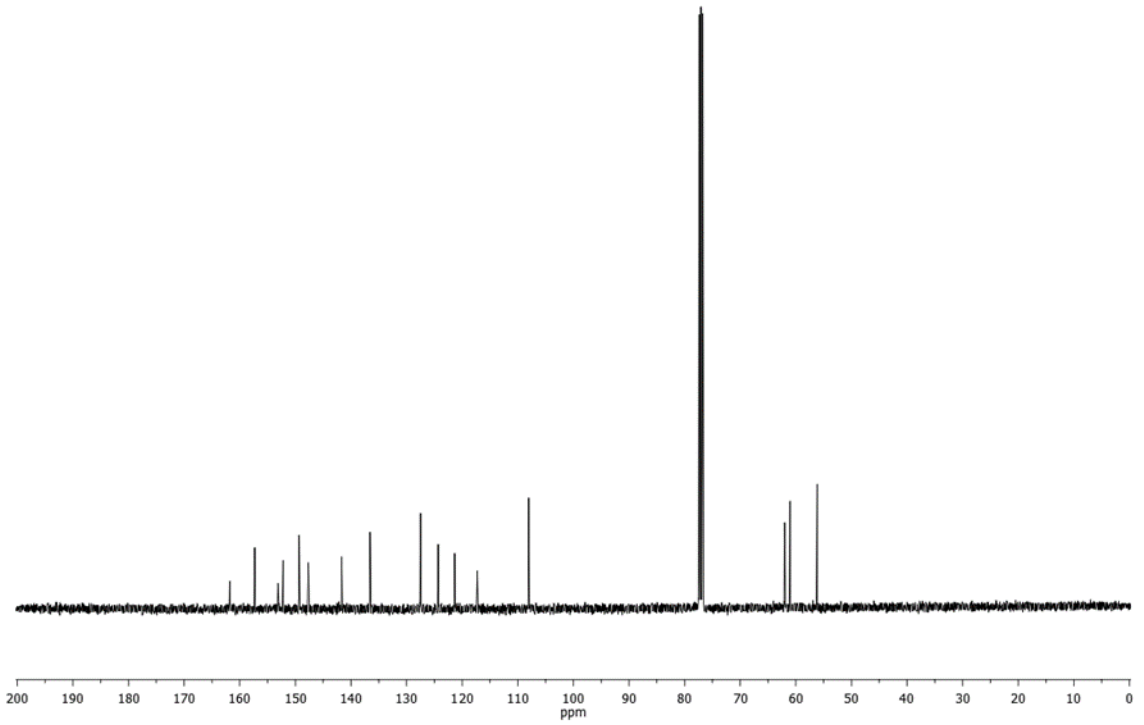

Figure S7. ^{13}C NMR spectra (100 MHz) in $CDCl_3$ of **3*E***

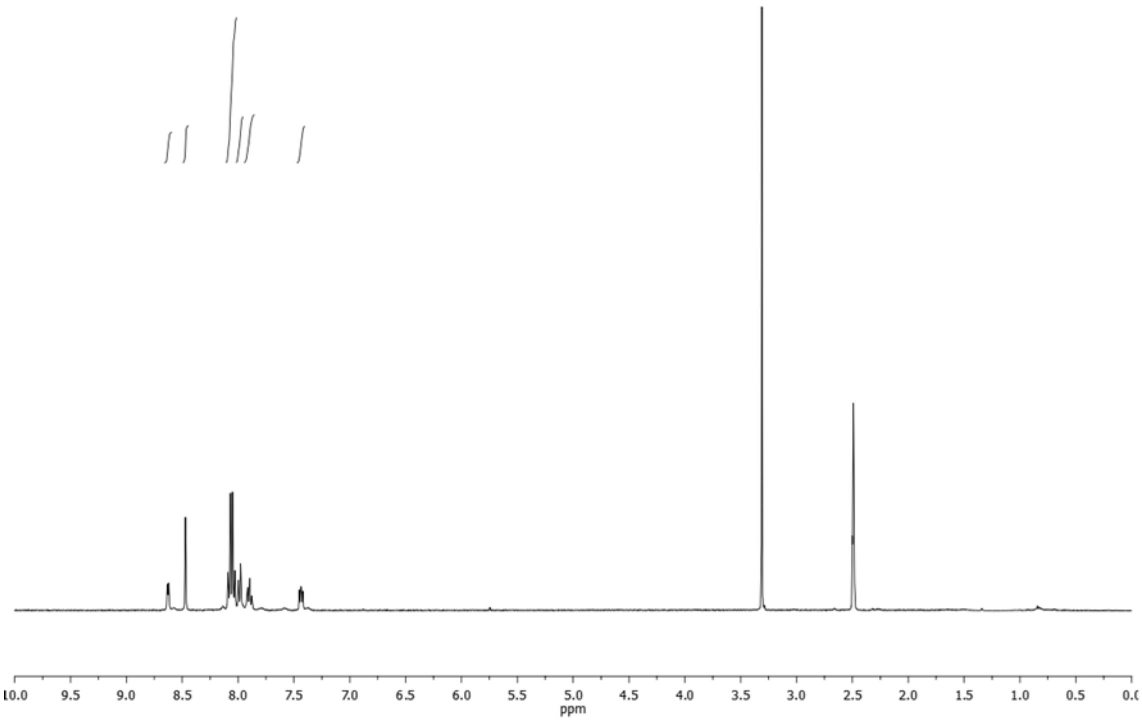

Figure S8. ^{1}H NMR spectra (400 MHz) in DMSO-d_6 of **4*E***

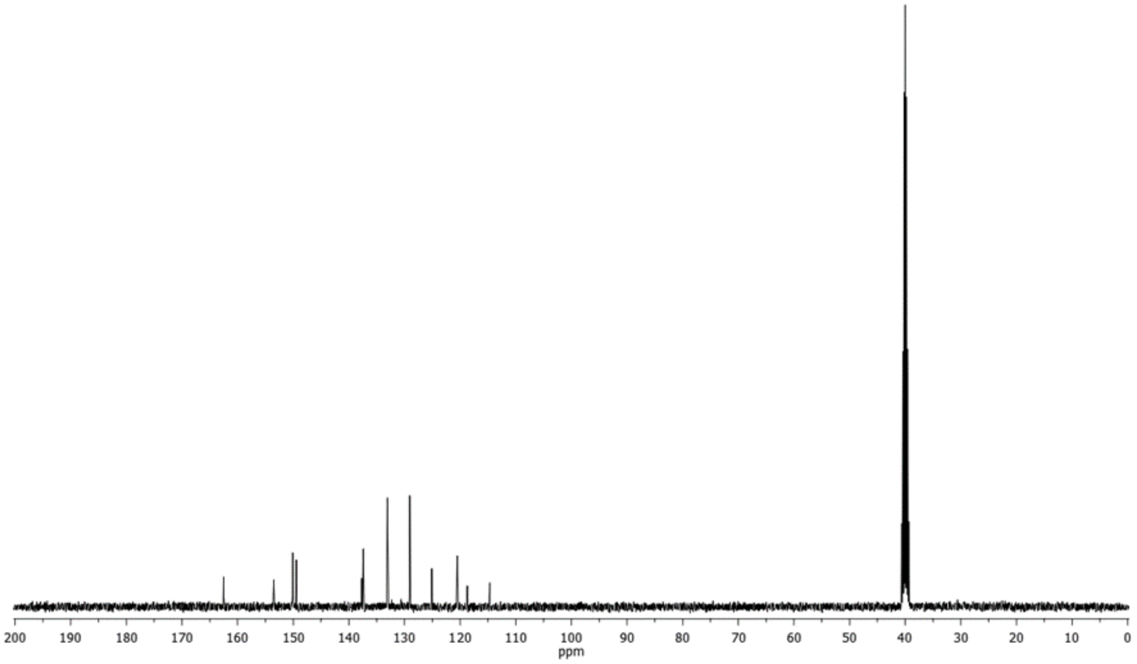

Figure S9. ^{13}C NMR spectra (100 MHz) in DMSO-d_6 of **4*E***

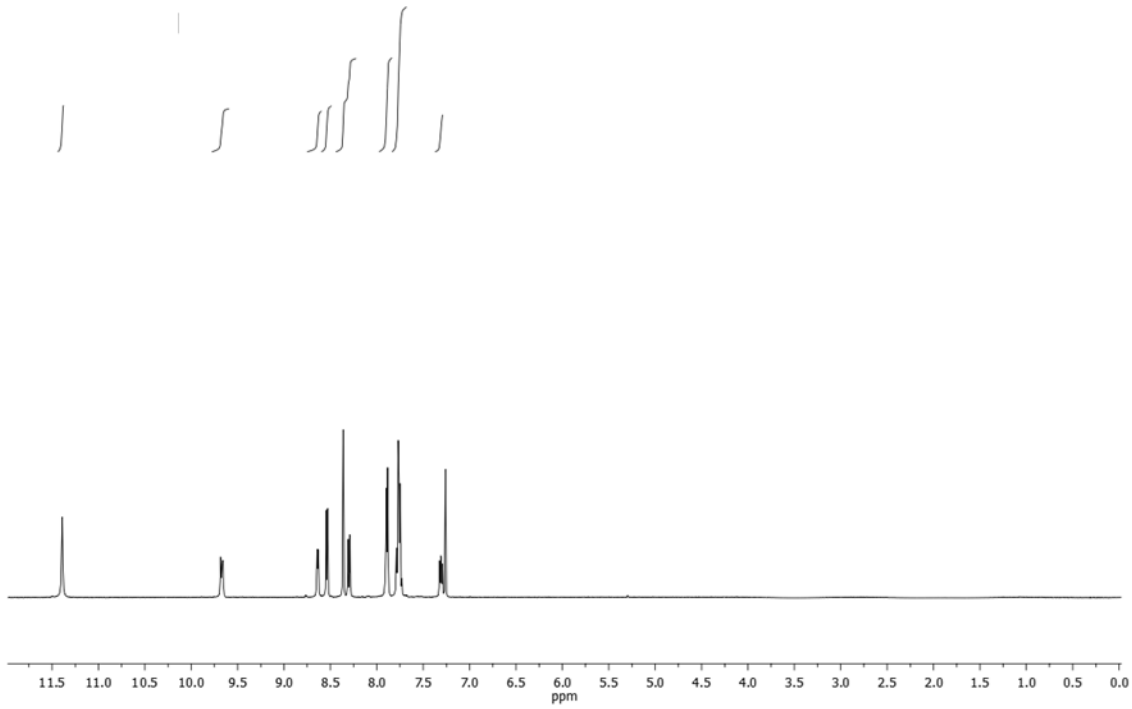

Figure S10. ^{1}H NMR spectra (400 MHz) in $CDCl_3$ of **5*E***

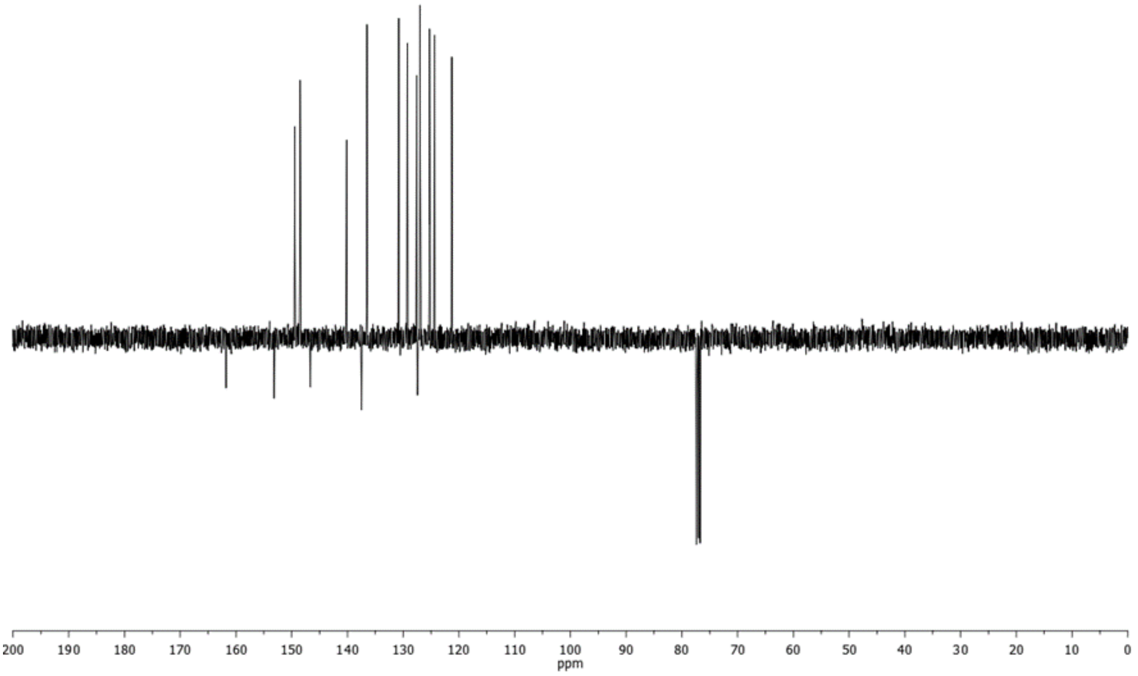

Figure S11. ^{13}C NMR spectra (100 MHz) in $CDCl_3$ of **5*E***

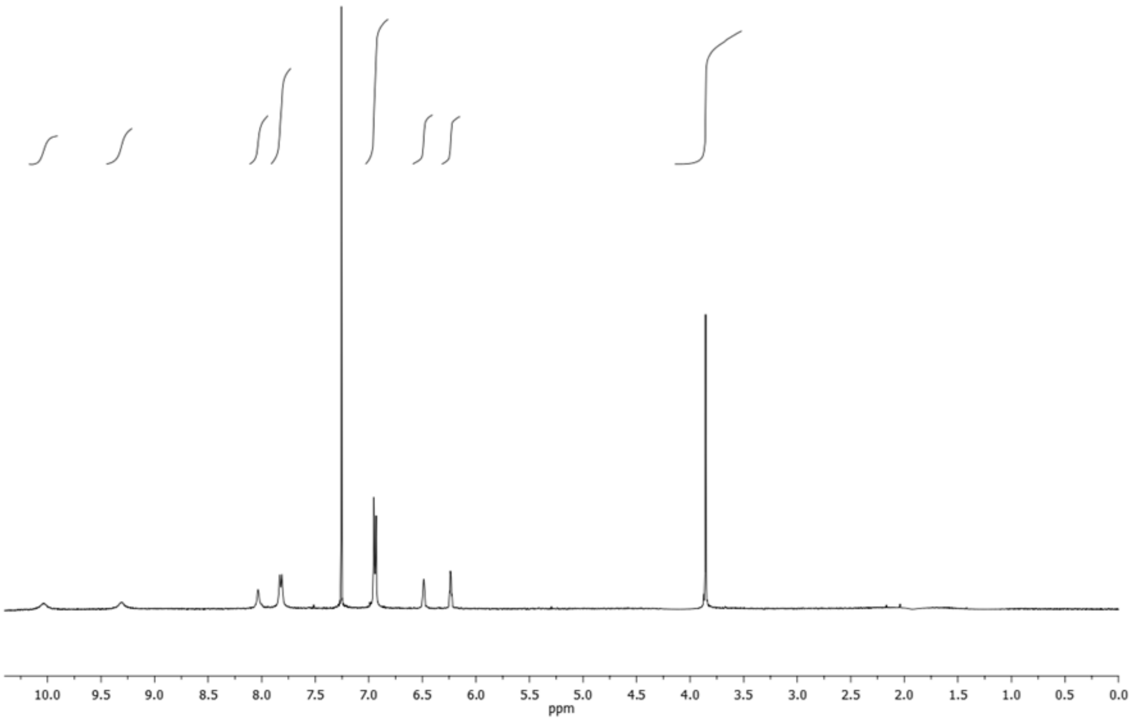

Figure S12. ^{1}H NMR spectra (400 MHz) in CD_3OD of **6*E***

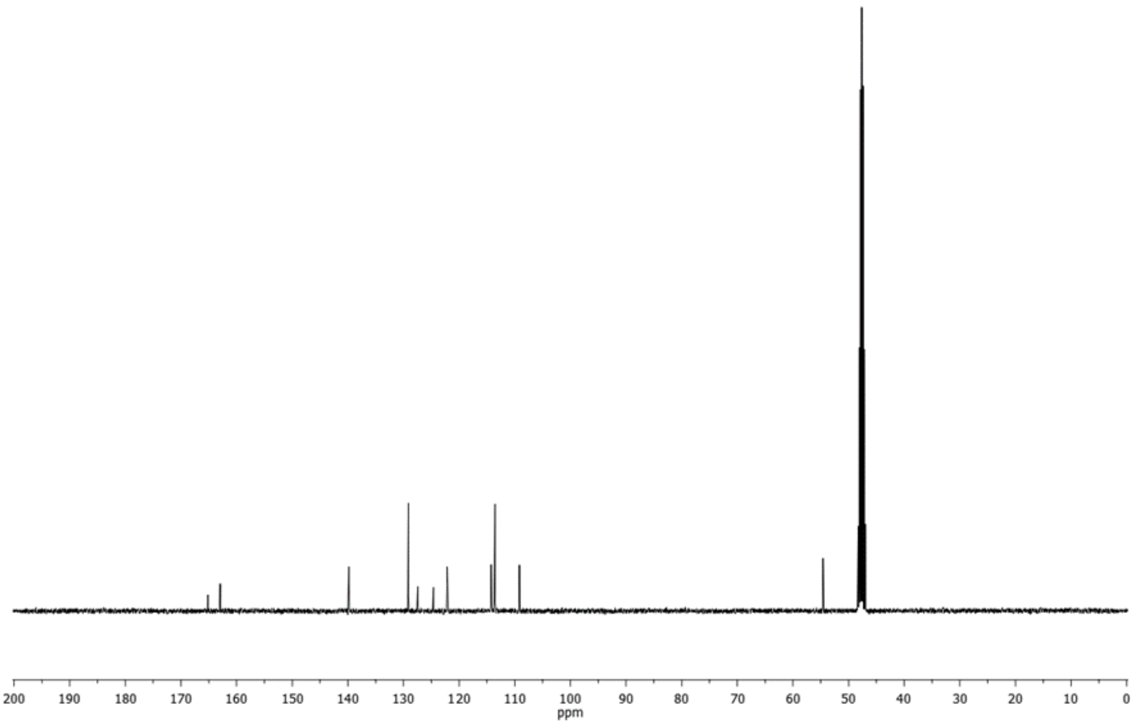

Figure S13. ^{13}C NMR spectra (100 MHz) in CD_3OD of **6*E***

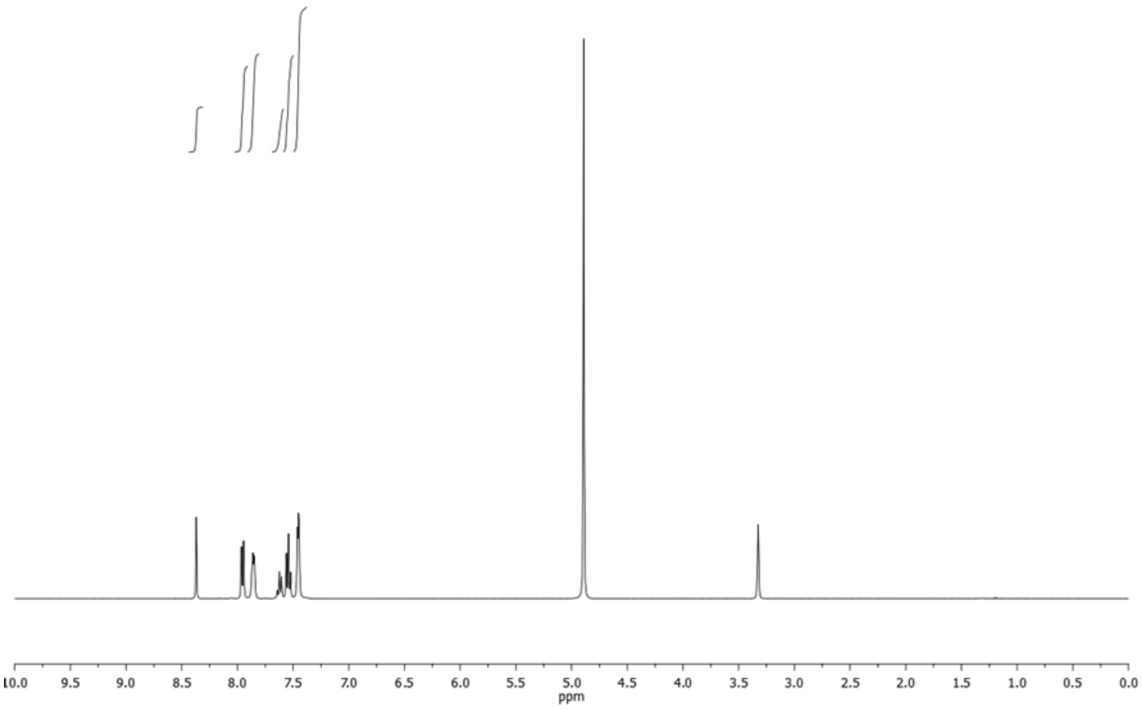

Figure S14. ^{1}H NMR spectra (400 MHz) in CD_3OD of **7*E***

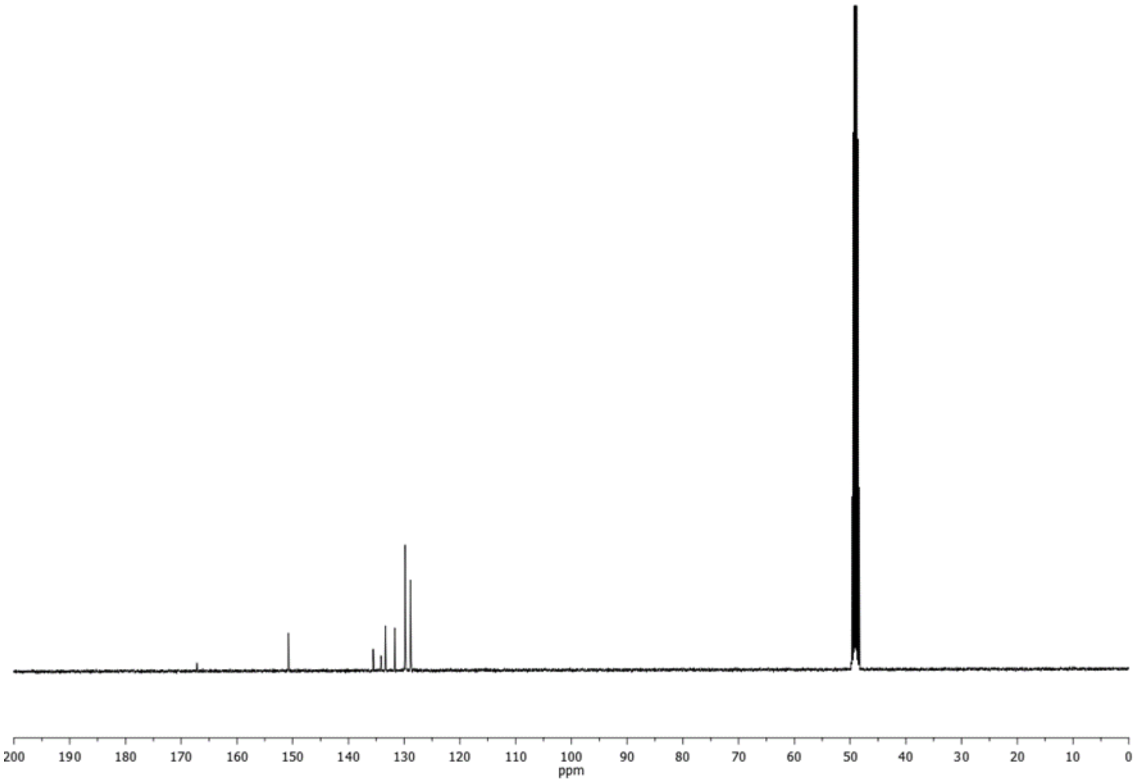

Figure S15. ^{13}C NMR spectra (100 MHz) in CD_3OD of **7*E***

3. Photophysical properties of acylhydrazones 1-7.

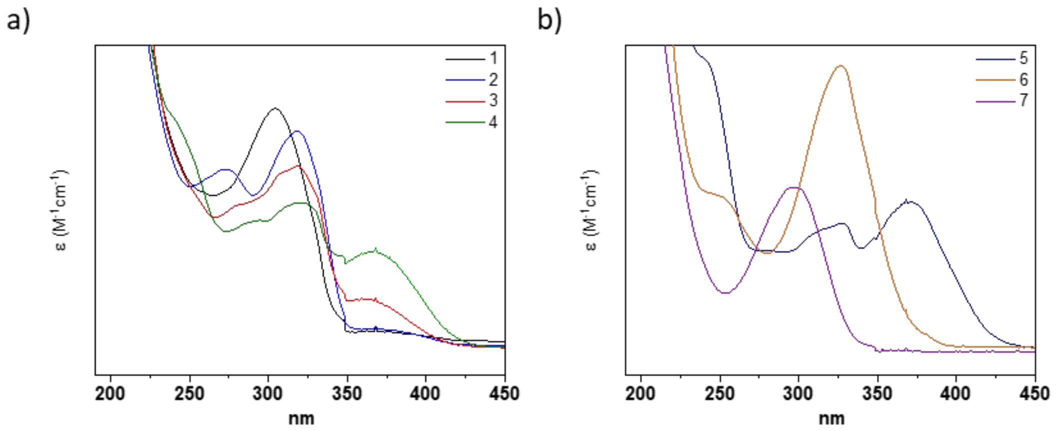

Figure S16. UV spectra of a) **1** (black), **2** (blue), **3** (red) and **4** (green) and b)), **5** (dark blue), **6** (orange) and **7** (purple), in CH_3OH at a concentration of 10^{-5} M.

4. Photophysical properties of acylhydrazones 1-7 in presence of metallic cations.

4.1. In acetonitrile solutions.

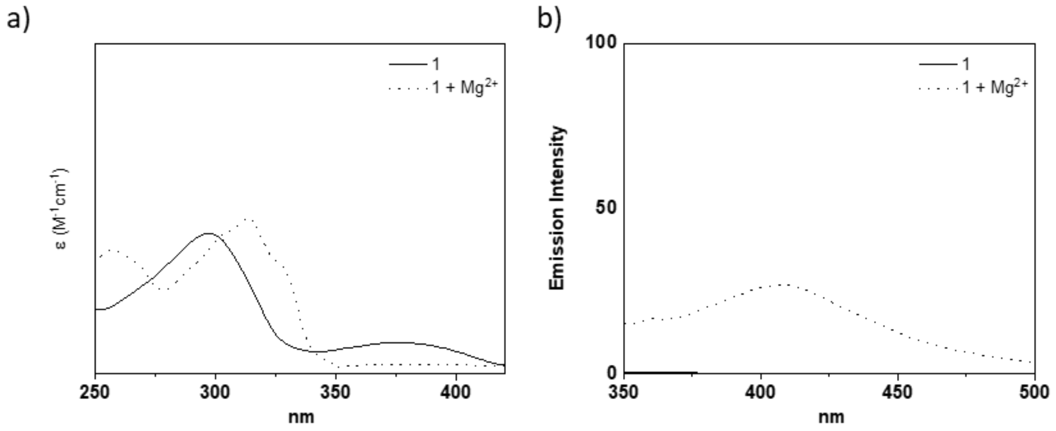

Figure S17. a) UV spectra and b) emission spectra (λ_{exc}=310 nm) of **1** (solid line) and **1+Mg^{2+}** (dotted line) in CH_3CN at a concentration of $5 \cdot 10^{-6}$ M.

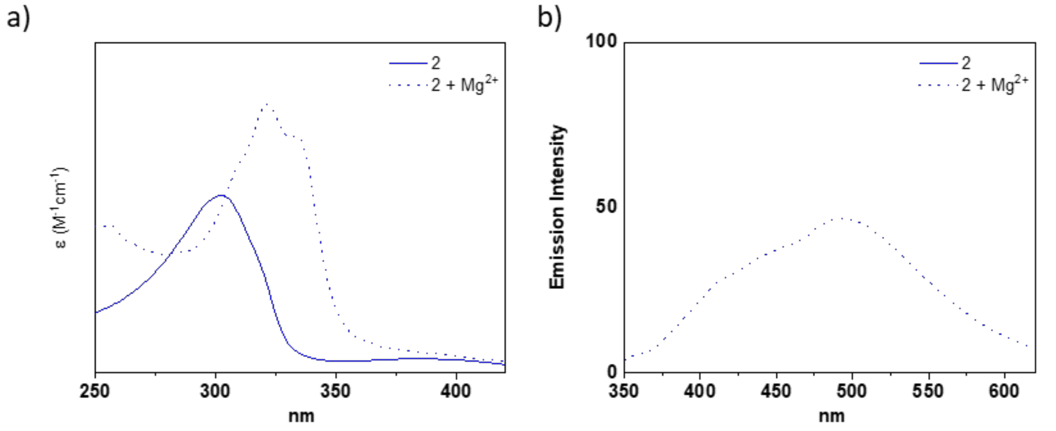

Figure S18. a) UV spectra and b) emission spectra (λ_{exc}=330 nm) of **2** (solid line) and **2+Mg^{2+}** (dotted line) in CH_3CN at a concentration of $5 \cdot 10^{-6}$ M.

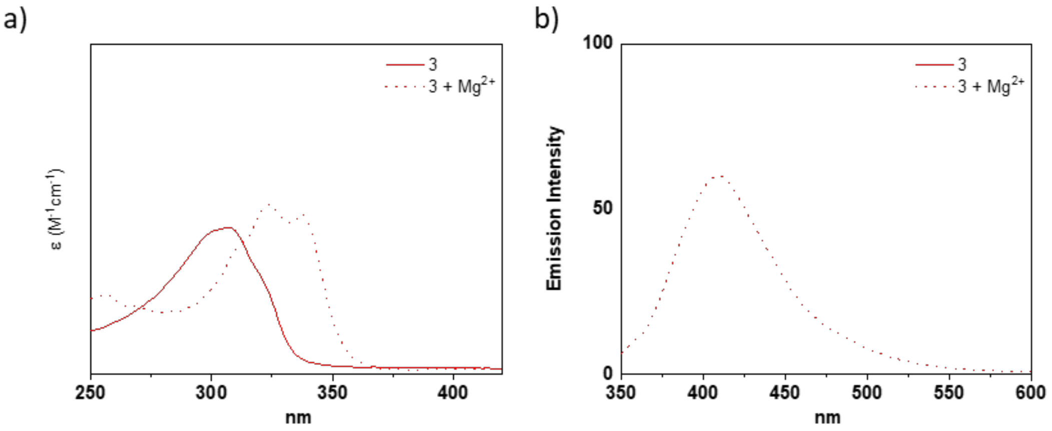

Figure S19. a) UV spectra and b) emission spectra (λ_{exc}=330 nm) of **3** (solid line) and **3+Mg**$^{2+}$ (dotted line) in CH_3CN at a concentration of $5 \cdot 10^{-6}$ M.

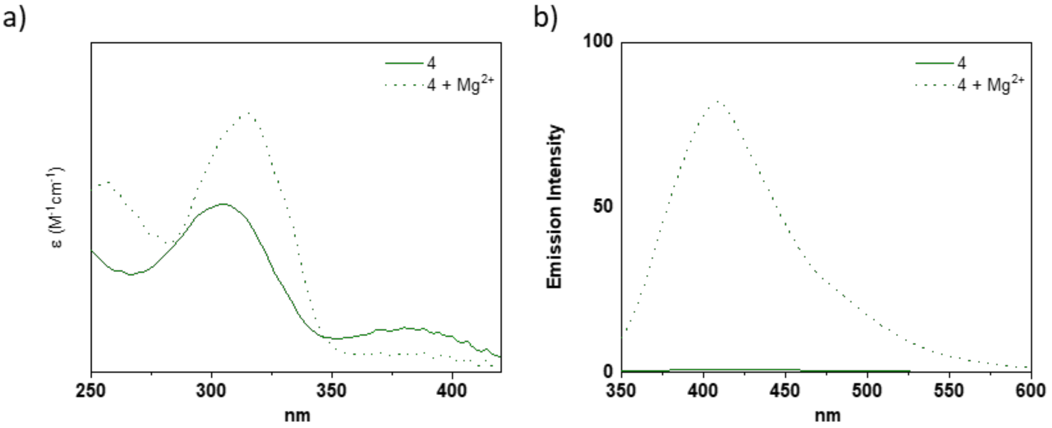

Figure S20. a) UV spectra and b) emission spectra (λ_{exc}=330 nm) of **4** (solid line) and **4+Mg**$^{2+}$ (dotted line) in CH_3CN at a concentration of $5 \cdot 10^{-6}$ M.

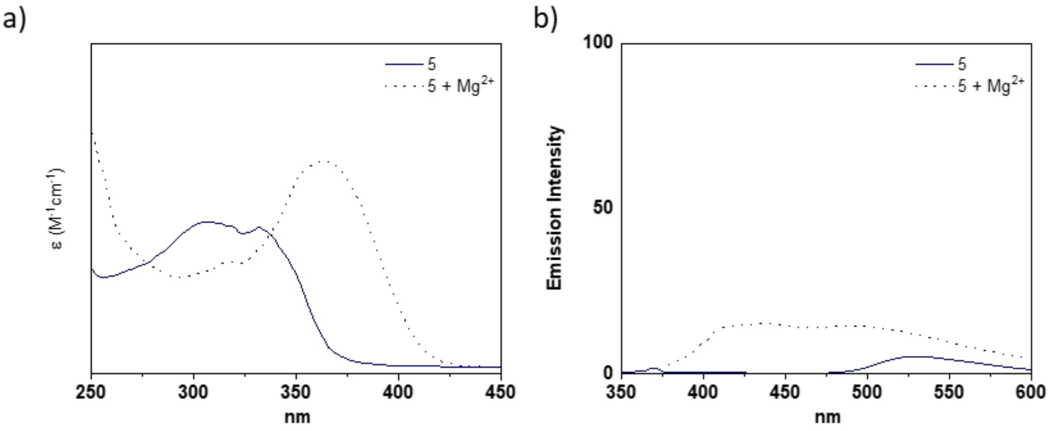

Figure S21. a) UV spectra and b) emission spectra (λ_{exc}=330 nm) of **5** (solid line) and **5+Mg**$^{2+}$ (dotted line) in CH_3CN at a concentration of $5 \cdot 10^{-6}$ M.

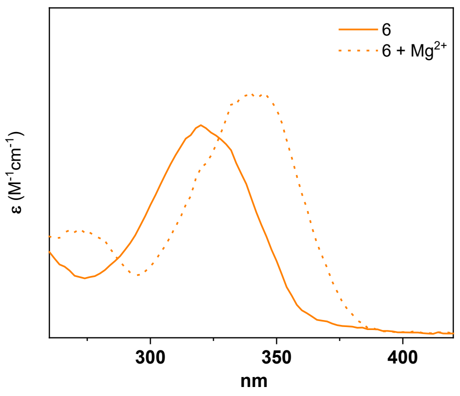

Figure S22. UV spectra of **6** (solid line) and **4+Mg^{2+}** (dotted line) in CH_3CN at a concentration of $5 \cdot 10^{-6}$ M.

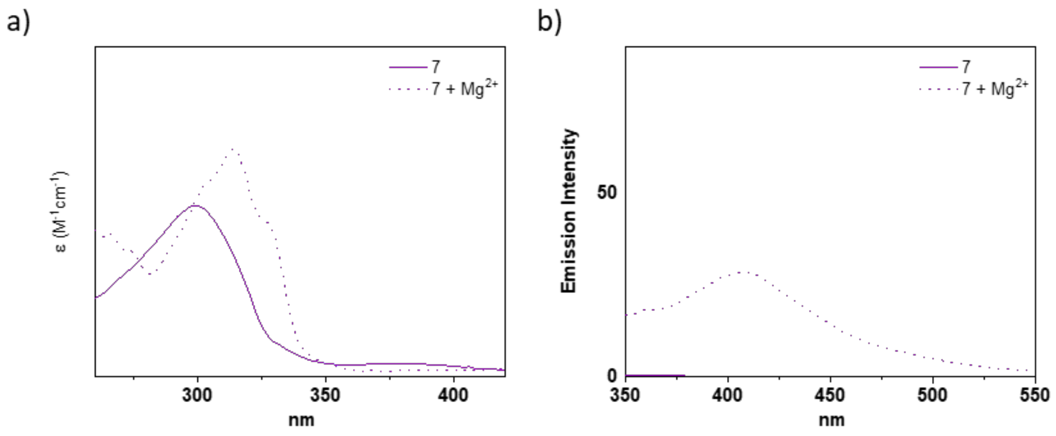

Figure S23. a) UV spectra and b) emission spectra (λ_{exc}=330 nm) of **7** (solid line) and **7+Mg^{2+}** (dotted line) in CH_3CN at a concentration of $5 \cdot 10^{-6}$ M.

4.2. In methanol solutions.

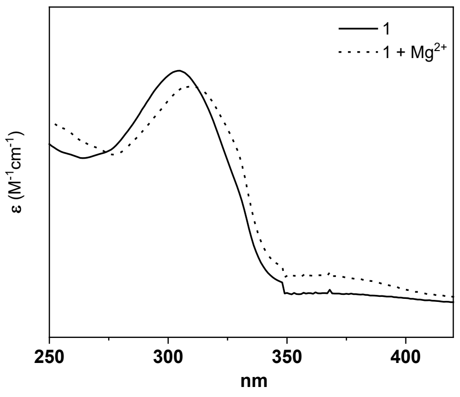

Figure S24. UV spectra of **1** (solid line) and **1+Mg^{2+}** (dotted line) in CH_3OH at a concentration of 10^{-5} M.

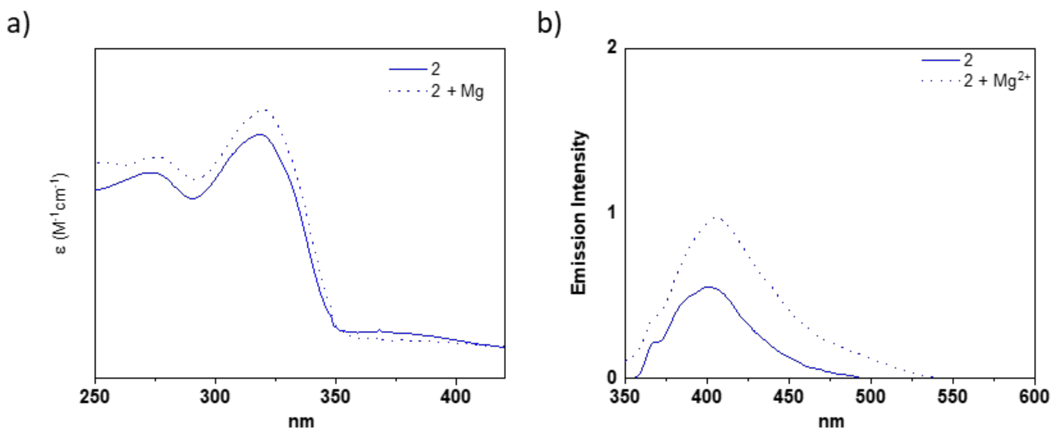

Figure S25. a) UV spectra and b) emission spectra (λ_{exc}=330 nm) of **2** (solid line) and **2+Mg^{2+}** (dotted line) in CH_3OH at a concentration of 10^{-5} M.

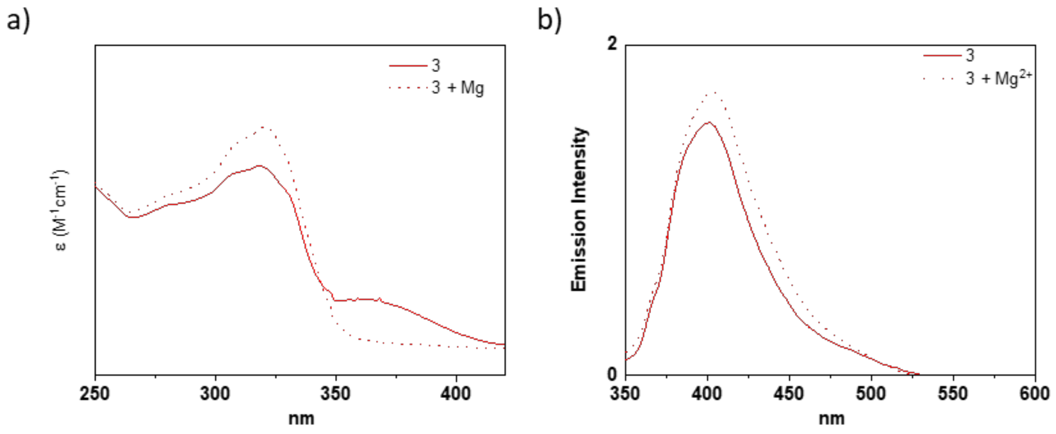

Figure S26. a) UV spectra and b) emission spectra (λ_{exc}=330 nm) of **3** (solid line) and **3+Mg^{2+}** (dotted line) in CH_3OH at a concentration of 10^{-5} M.

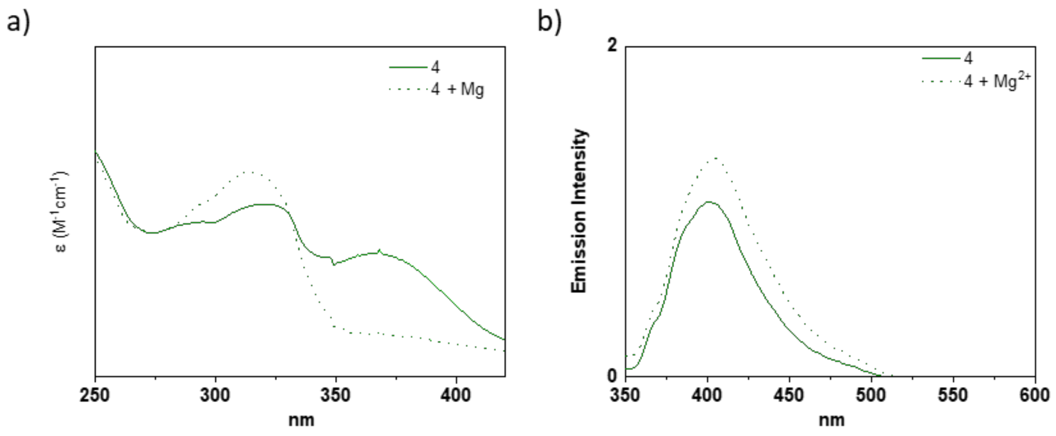

Figure S27. a) UV spectra and b) emission spectra (λ_{exc}=330 nm) of **4** (solid line) and **4+Mg^{2+}** (dotted line) in CH_3OH at a concentration of 10^{-5} M.

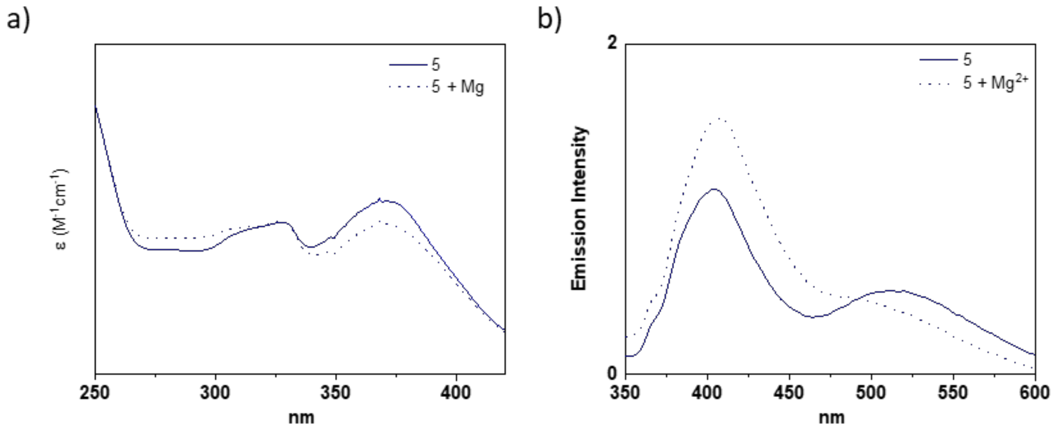

Figure S28. a) UV spectra and b) emission spectra (λ_{exc}=330 nm) of **5** (solid line) and **5+Mg^{2+}** (dotted line) in CH_3OH at a concentration of 10^{-5} M.

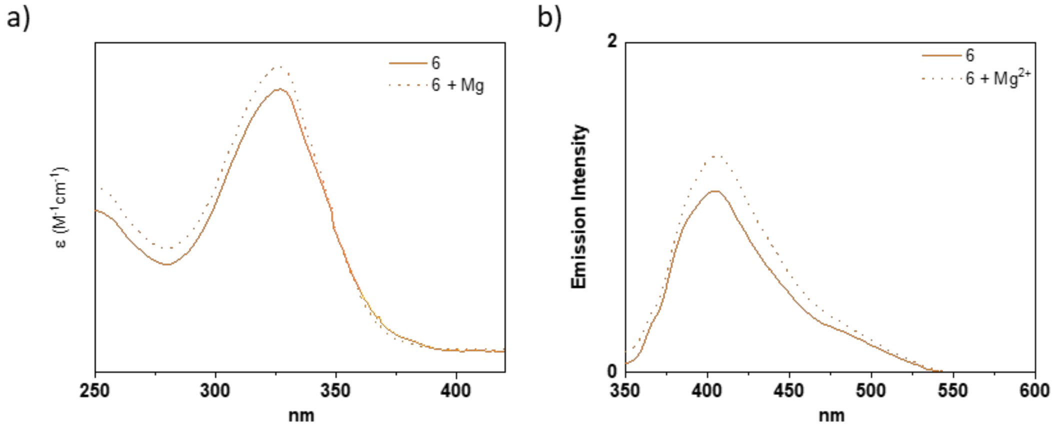

Figure S29. a) UV spectra and b) emission spectra (λ_{exc}=330 nm) of **6** (solid line) and **6+Mg^{2+}** (dotted line) in CH_3OH at a concentration of 10^{-5} M.

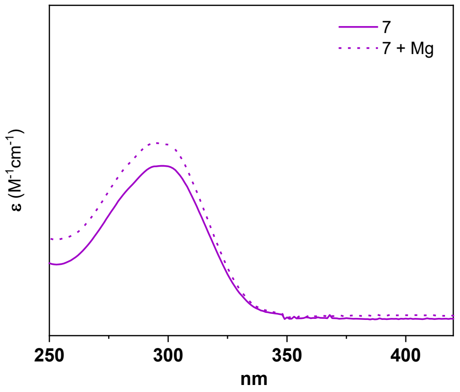

Figure S30. UV spectra of **7** (solid line) and **1+Mg^{2+}** (dotted line) in CH_3OH at a concentration of 10^{-5} M.

5. Photochemical properties of acylhydrazones 1-7.

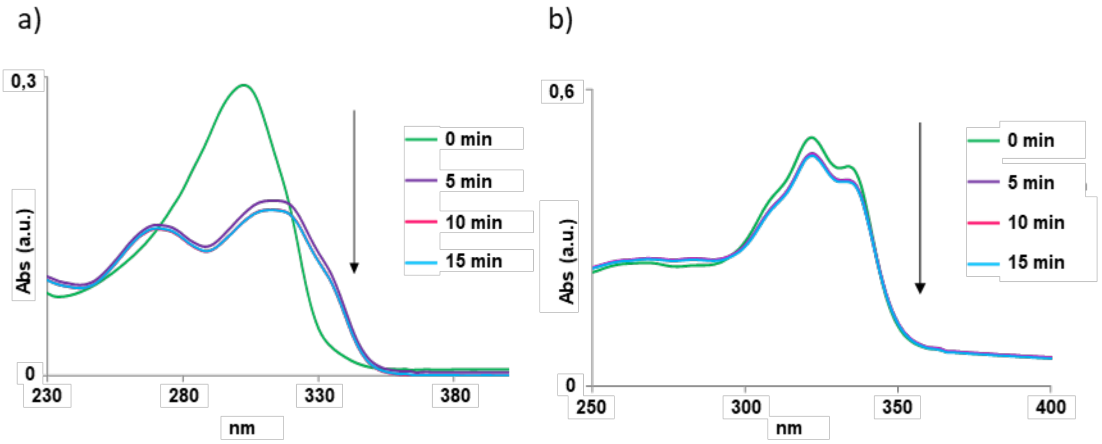

Figure S31. a) UV spectra of **2*E*** (green) in CH_3CN at a concentration of 10^{-5} M and the solution after 5, 10 and 15 minutes of UV irradiation. b) UV spectra of **2*E*+Mg^{2+}** (green) in CH_3CN at a concentration of 10^{-5} M and the solution after 5, 10 and 15 minutes of UV irradiation.

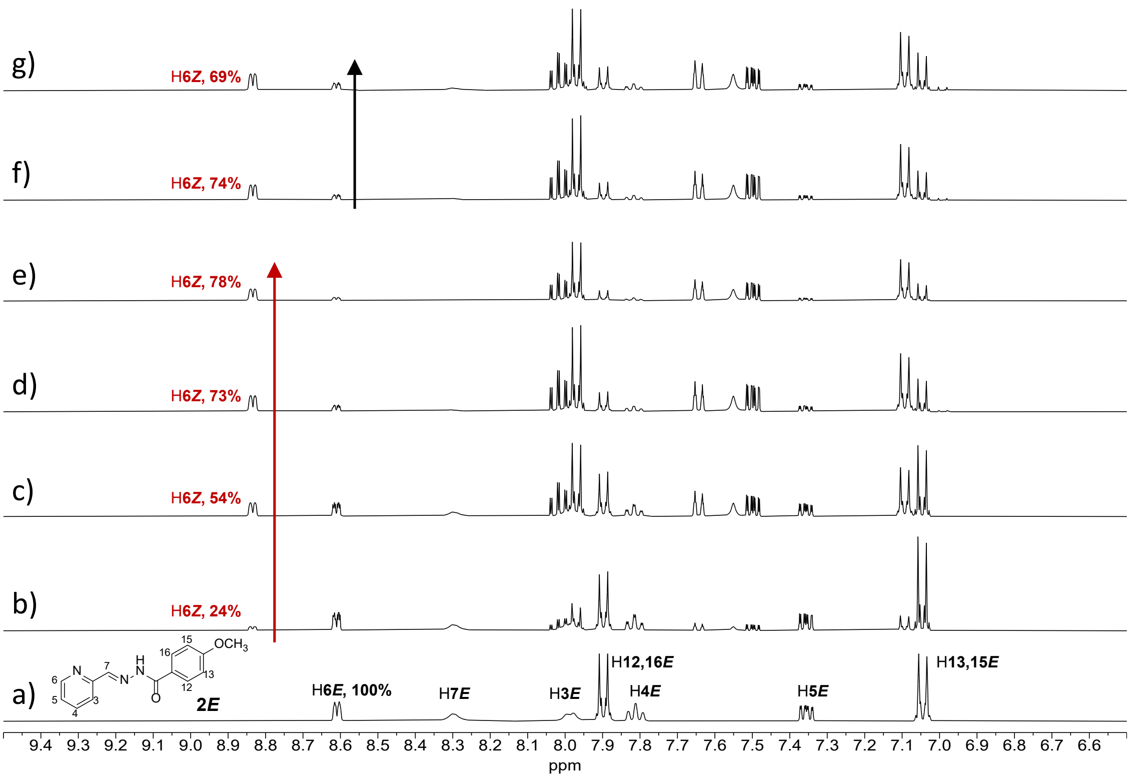

Figure S32. ^{1}H NMR spectra (400 MHz) in CD_3CN of a) **2*E*** and the solution after b) 5, c) 15, d) 30, e) 45, f) 75 and g) 120 minutes of UV irradiation. The initial concentration of **2*E*** is 13 mM.

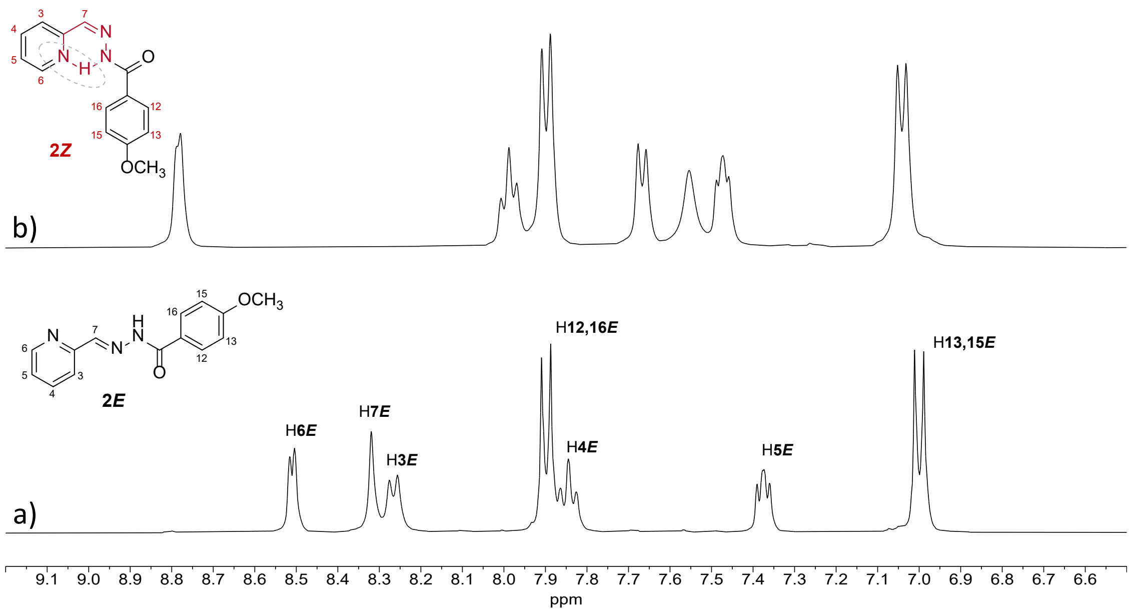

Figure S33. ^{1}H NMR spectra (400 MHz) in CD_3OD of a) ***2E*** and b) the solution after 45 minutes of UV irradiation (formation of 100% ***2Z***). The initial concentration of ***2E*** is 13 mM.

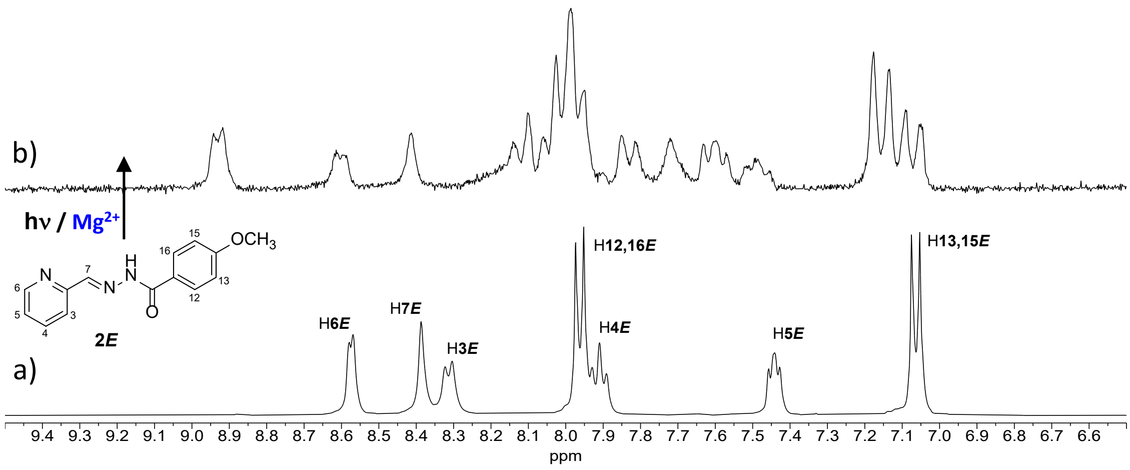

Figure S34. ^{1}H NMR spectra (400 MHz) in CD_3OD of a) ***2E*** and b) a solution of ***2E*** + 10 eq. of $MgClO_4$ after 45 minutes of UV irradiation. The initial concentration of ***2E*** is 13 mM.

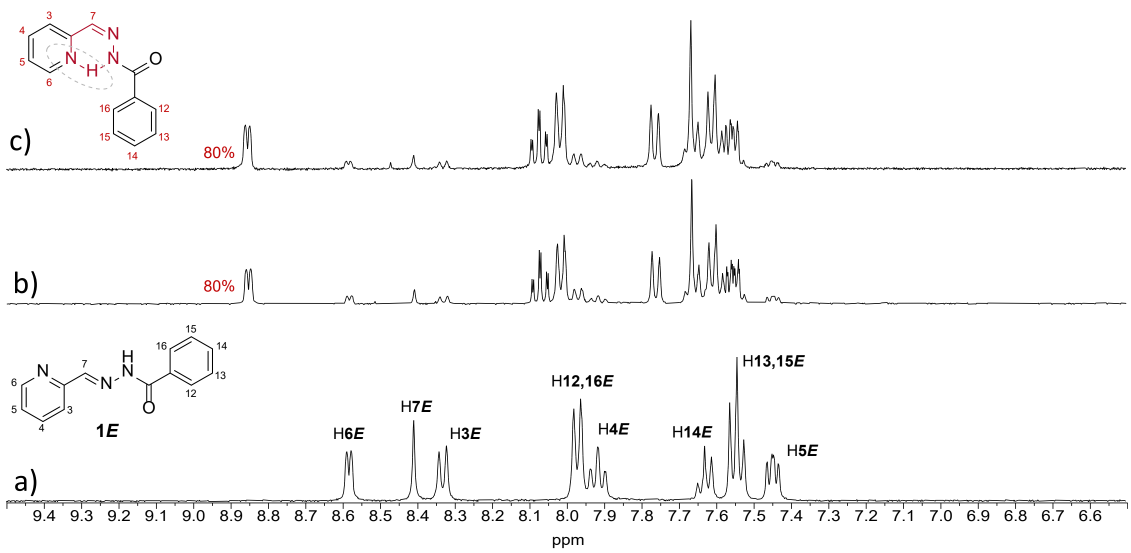

Figure S35. ^{1}H NMR spectra (400 MHz) in CD_3OD of a) **1*E***, b) the solution after 45 minutes and c) the solution after 120 minutes of UV irradiation. The initial concentration of **1*E*** is 13 mM.

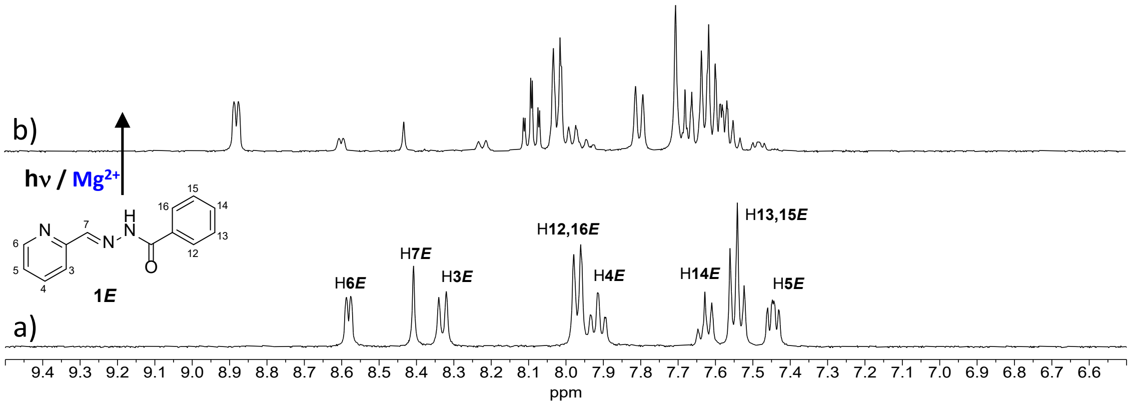

Figure S36. ^{1}H NMR spectra (400 MHz) in CD_3OD of a) **1*E*** and b) **1*E*** + 10 eq. of $MgClO_4$ after 45 minutes of UV irradiation. The initial concentration of **1*E*** is 13 mM.

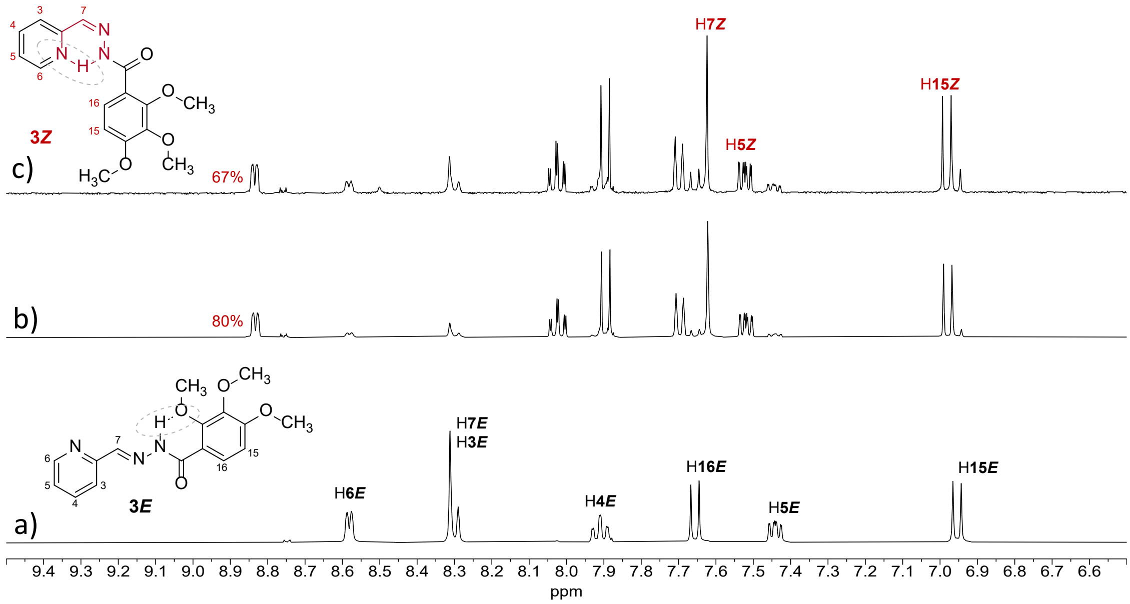

Figure S37. ^{1}H NMR spectra (400 MHz) in CD_3OD of a) **3*E***, b) the solution after 45 minutes and c) the solution after 120 minutes of UV irradiation. The initial concentration of **3*E*** is 13 mM.

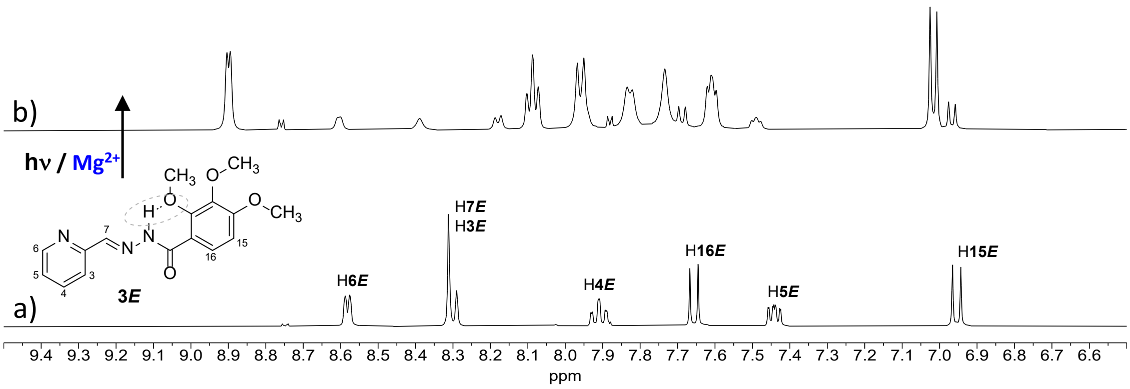

Figure S38. ^{1}H NMR spectra (400 MHz) in CD_3OD of a) **3*E*** and b) **3*E*** + 10 eq. of $MgClO_4$ after 45 minutes of UV irradiation. The initial concentration of **3*E*** is 13 mM.

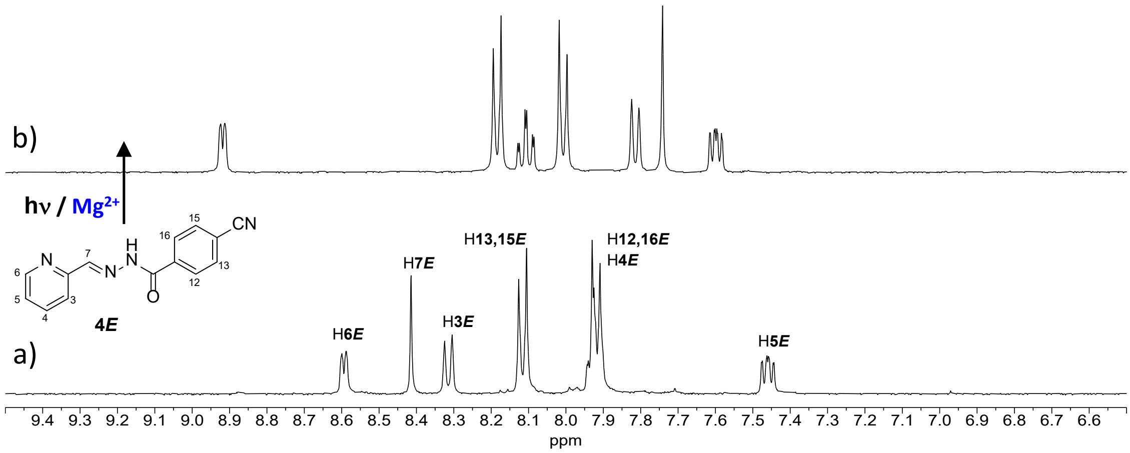

Figure S39. ^{1}H NMR spectra (400 MHz) in CD_3OD of a) ***4E*** and b) ***4E*** + 10 eq. of $MgClO_4$ after 45 minutes of UV irradiation. The initial concentration of ***3E*** is 13 mM.

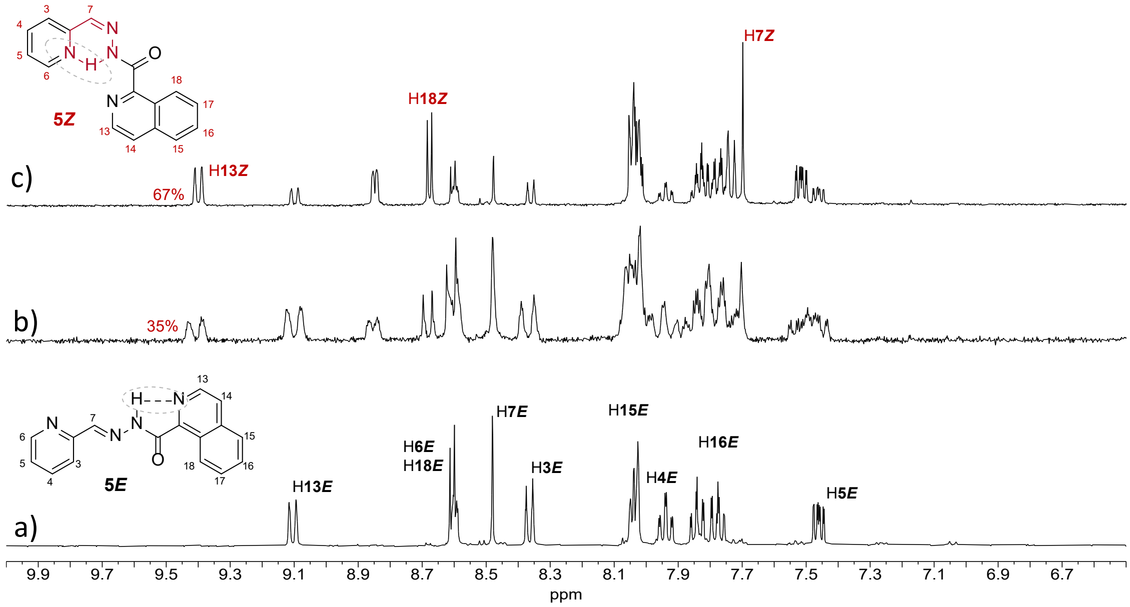

Figure S40. ^{1}H NMR spectra (400 MHz) in CD_3OD of a) ***5E***, b) the solution after 45 minutes and c) the solution after 120 minutes of UV irradiation. The initial concentration of ***5E*** is 13 mM.

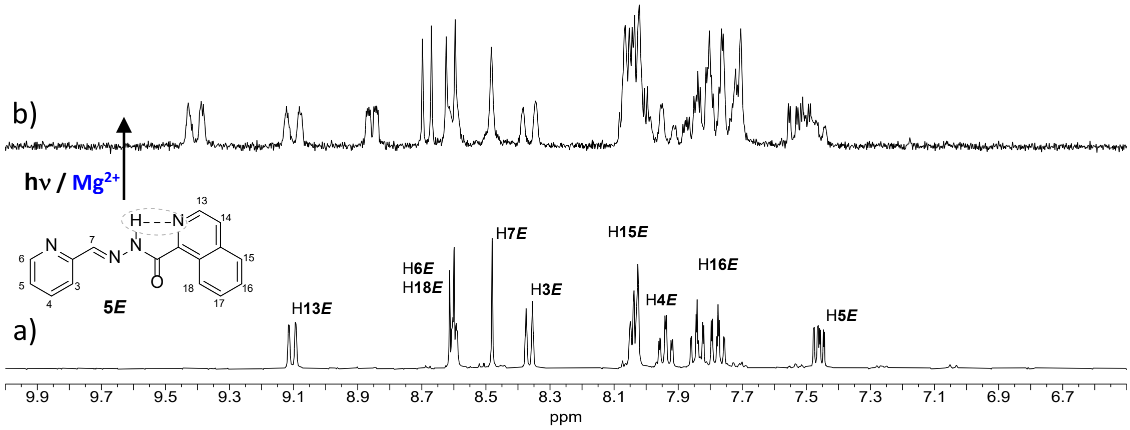

Figure S41. ^{1}H NMR spectra (400 MHz) in CD_3OD of a) ***5E*** and b) ***5E*** + 10 eq. of $MgClO_4$ after 45 minutes of UV irradiation. The initial concentration of ***5E*** is 13 mM.

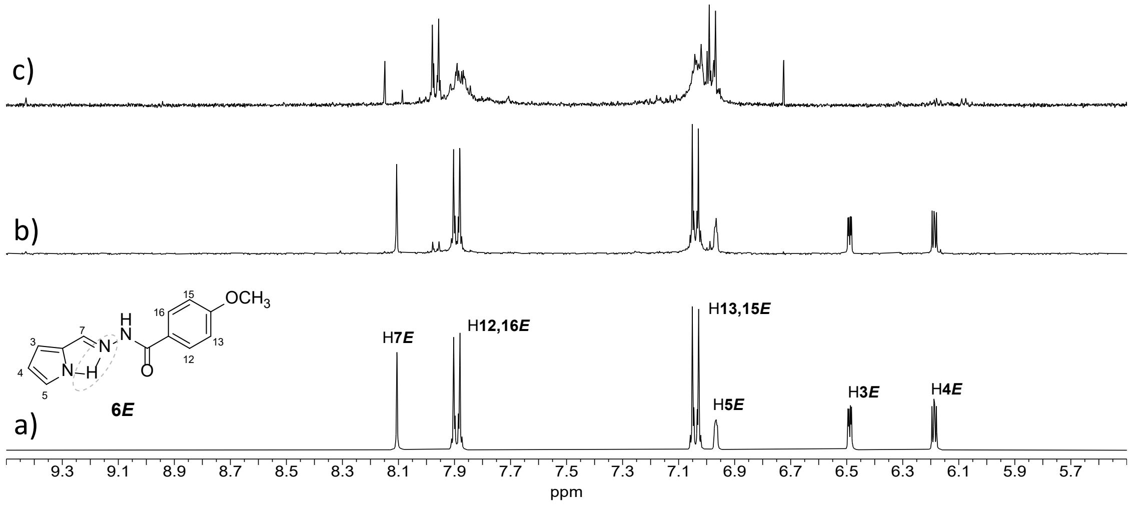

Figure S42. ^{1}H NMR spectra (400 MHz) in CD_3OD of a) **6*E***, b) the solution after 45 minutes and c) the solution after 120 minutes of UV irradiation. The initial concentration of **6*E*** is 13 mM.

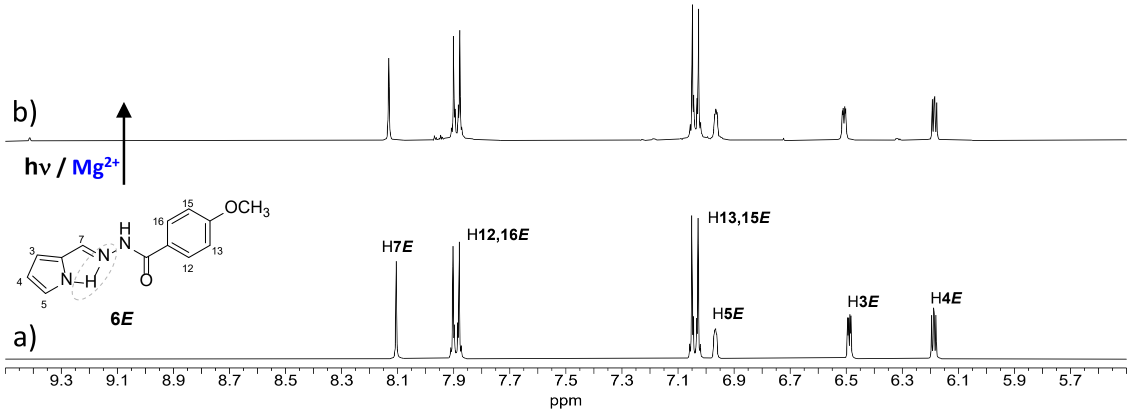

Figure S43. ^{1}H NMR spectra (400 MHz) in CD_3OD of a) ***6E*** and b) ***6E*** + 10 eq. of $MgClO_4$ after 45 minutes of UV irradiation. The initial concentration of ***6E*** is 13 mM.

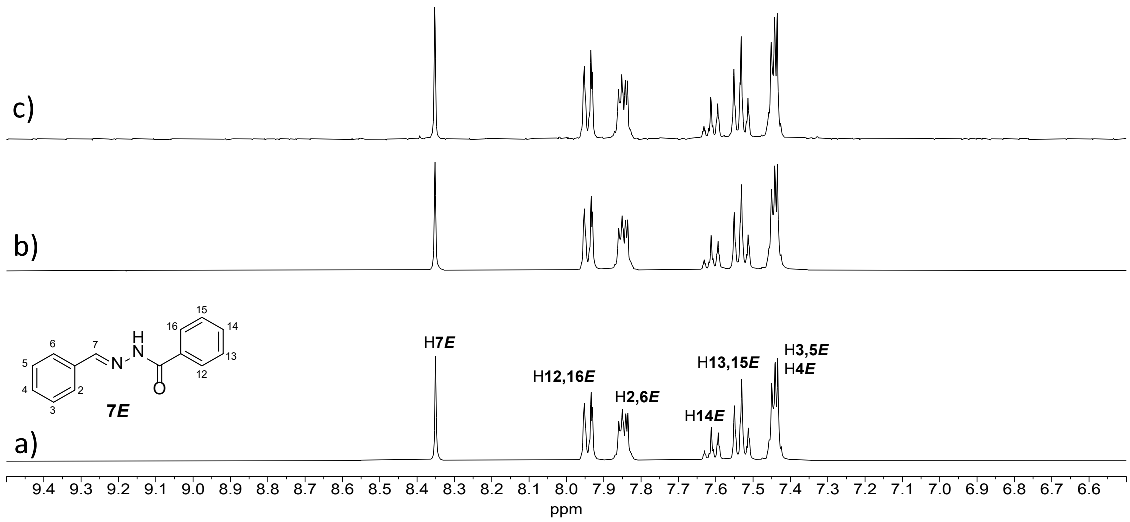

Figure S44. ^{1}H NMR spectra (400 MHz) in CD_3OD of a) ***7E***, b) the solution after 45 minutes and c) the solution after 120 minutes of UV irradiation. The initial concentration of ***7E*** is 13 mM.

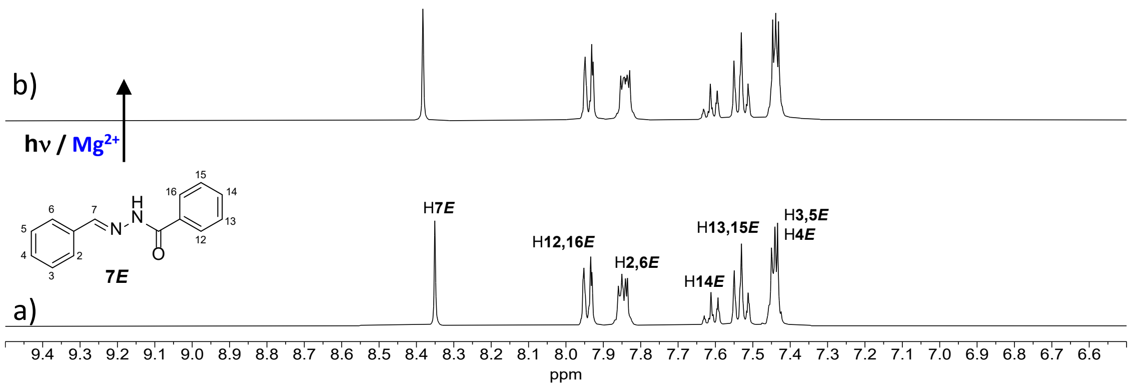

Figure S45. ^{1}H NMR spectra (400 MHz) in CD_3OD of a) ***7E*** and b) ***7E*** + 10 eq. of $MgClO_4$ after 45 minutes of UV irradiation. The initial concentration of ***7E*** is 13 mM.

6. DFT and TD-DFT calculations.

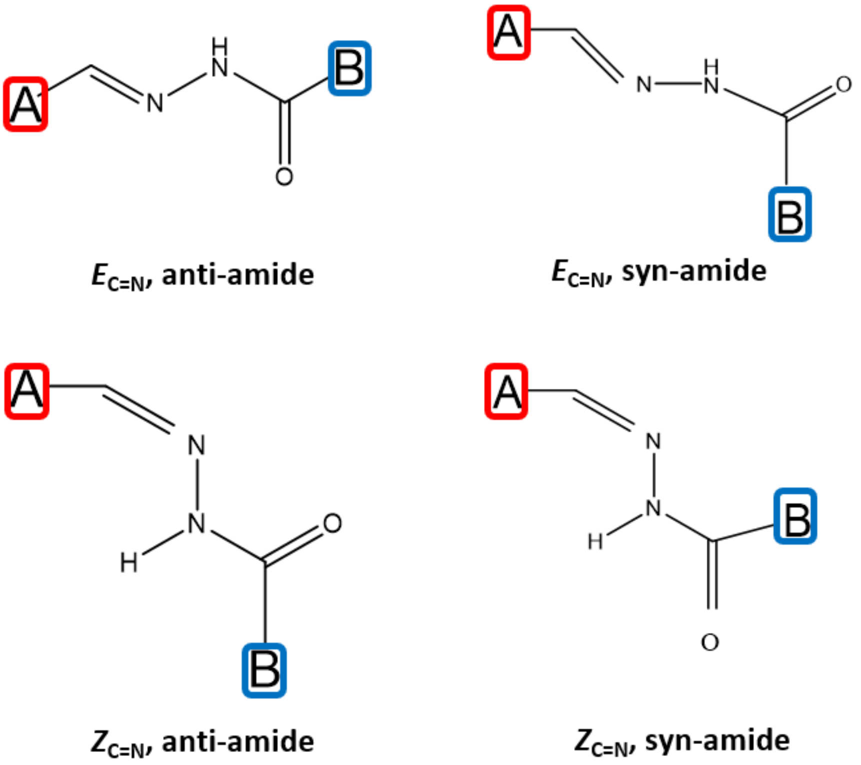

Figure S46. Different conformations and configurations in acylhydrazone derivatives.

Table S1. DFT-calculated relative energies (kcal/mol) of the *anti*- and *syn*-amide configurations of the **1-7** (*E*)-isomers.

Compounds	Relative Energy (kcal/mol)			
	PCM-CAM-B3LYP/6-31G**		PCM-B3LYP/6-31G**	
	E, anti	*E*, syn	*E*, anti	*E*, syn
**1**	0	0.96	0	0.73
**2**	0	0.85	0	0.56
**3**	0	4.69	0	4.22
**4**	0	0.53	0	0.25
**5**	0	5.56	0	5.70
**6**	0	0.96	0	0.71
**7**	0	0.95	0	0.71

Table S2 DFT-calculated relative energies (kcal/mol) of the *anti*- and *syn*-amide configurations of the **1**-**7** (*Z*)-isomers.

Compounds	Relative Energy (kcal/mol)			
	PCM-CAM-B3LYP/6-31G**		PCM-B3LYP/6-31G**	
	Z, anti	*Z*, syn	*Z*, anti	*Z*, syn
**1**	0	1.79	0	1.34
**2**	0	1.73	0	1.25
**3**	0	3.43	0	2.46
**4**	0	1.39	0	0.92
**5**	0	5.61	0	5.72
**6**	0	0.13	0	-0.14
**7**	0	0.61	0	0.29

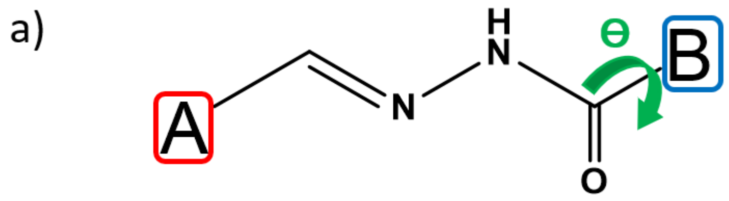

Compound	1*E*	2*E*	3*E*	4*E*	5*E*	6*E*	7*E*
ϴ (°)	25	21	168	27	180	22	25

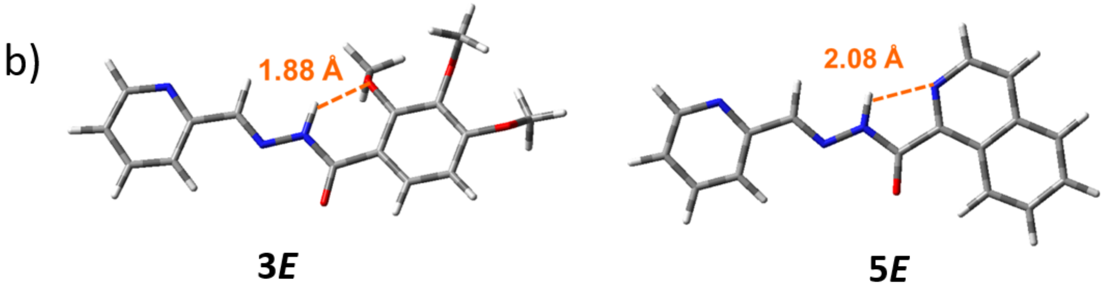

Figure S47. (a) DFT-calculated (PCM-B3LYP/6-31G** using acetonitrile as solvent) dihedral angles (°) between the B-ring and the hydrazide group of **1**-**7** (*E*)-isomers, and (b) top views of optimized geometries of **3** and **5** (*E*)-isomers with the intramolecular H-bond distances shown in orange.

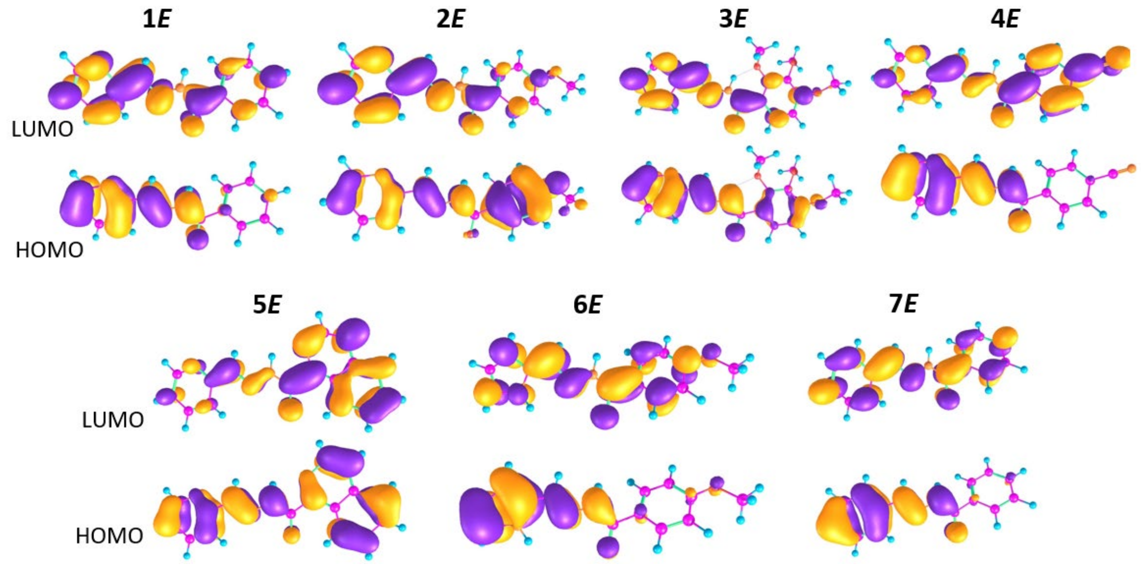

Figure S48. DFT-calculated frontier molecular orbital topologies of **1**-**7** (*E*)-isomers, at the PCM-CAM-B3LYP/6-31G** level using acetonitrile as solvent.

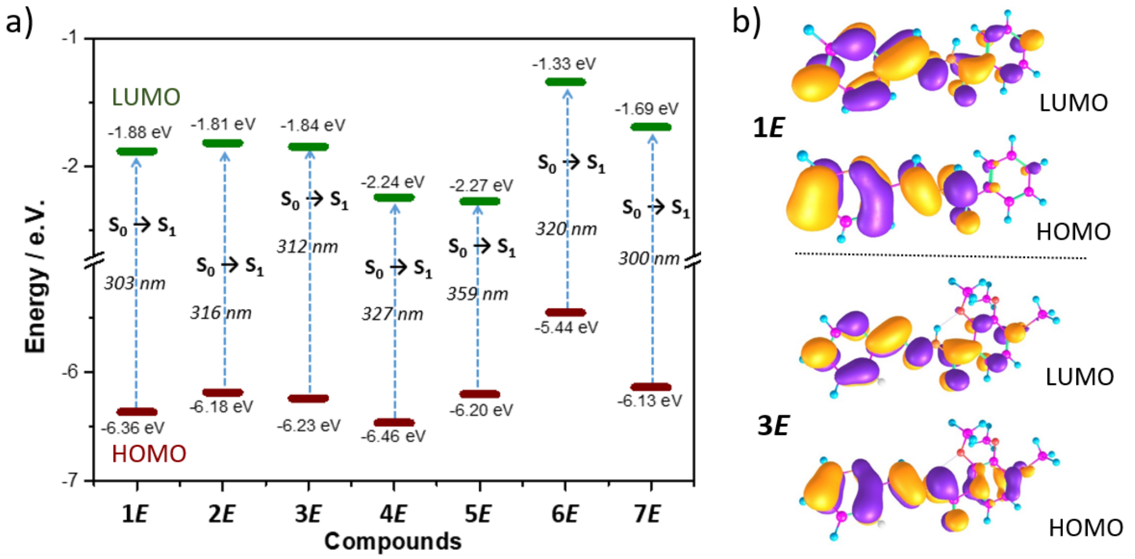

Figure S49. (a) DFT-calculated (PCM-B3LYP/6-31G** using acetonitrile as solvent) HOMO and LUMO energies of **1**-**7** *(E)*-isomers. TD-DFT calculated electronic absorption wavelengths (nm) for the lowest-energy electronic transitions are also shown. (b) The topologies of the HOMO and LUMO of **1*E*** and **3*E*** (for the rest of compounds see Figure S50).

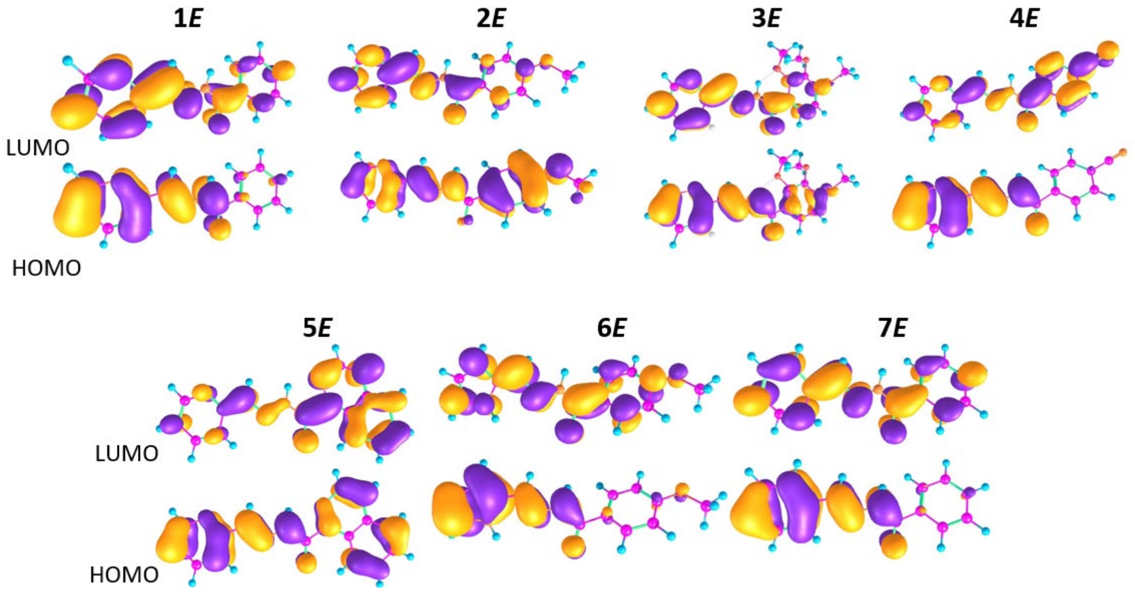

Figure S50. DFT-calculated frontier molecular orbital topologies of **1**-**7** (*E*)-isomers, at the PCM-B3LYP/6-31G** level using acetonitrile as solvent.

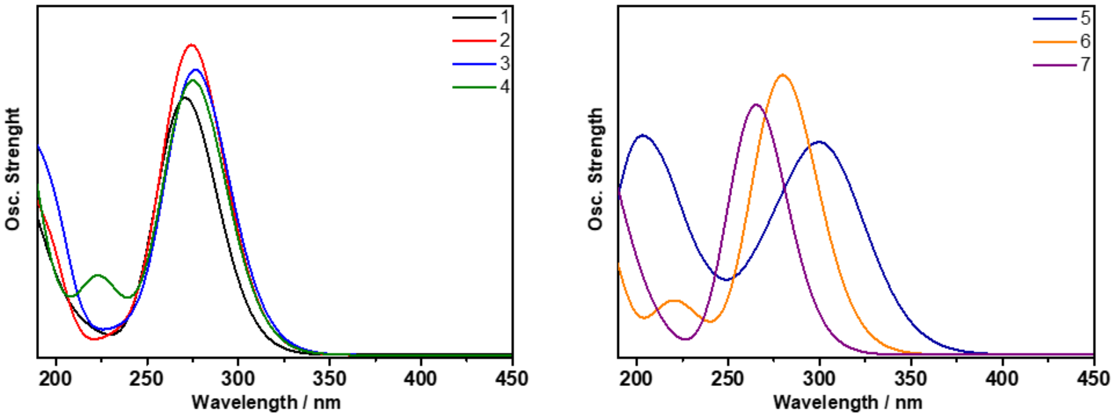

Figure S51. TD-DFT-calculated electronic absorption spectra for **1**-**7** (*E*)-isomers, at the PCM-CAM-B3LYP/6-31G** level using acetonitrile as solvent.

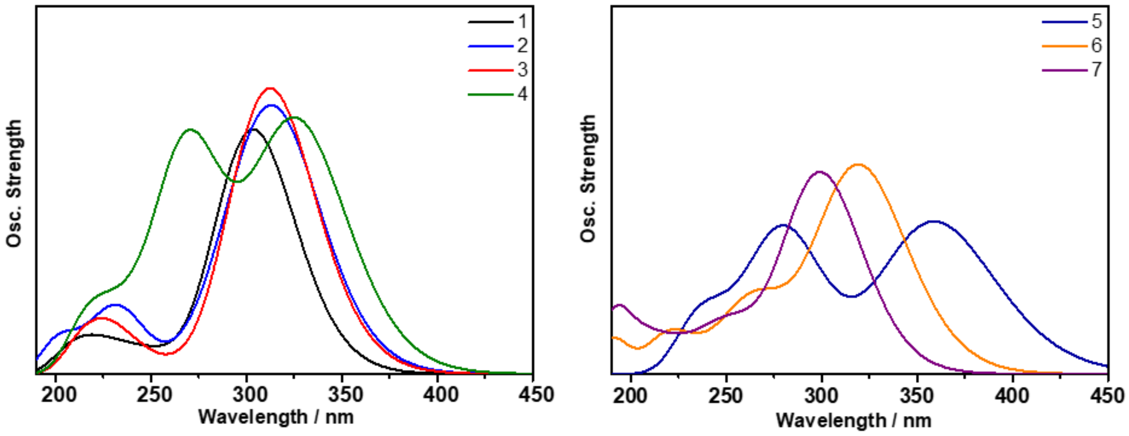

Figure S52. TD-DFT-calculated electronic absorption spectra for **1**-**7** (*E*)-isomers, at the PCM-B3LYP/6-31G** level using acetonitrile as solvent.

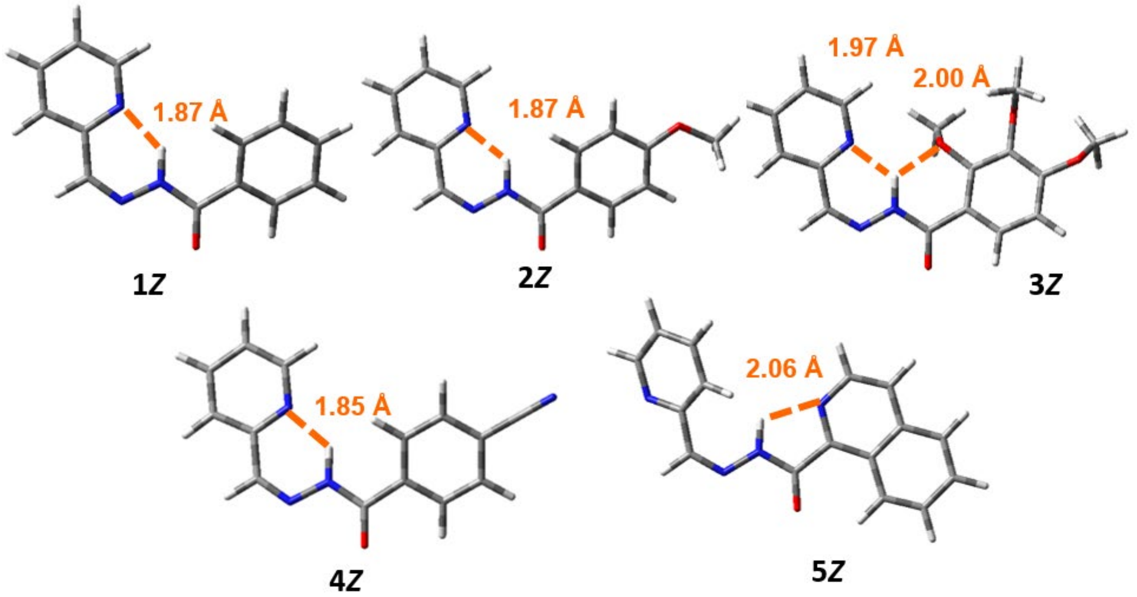

Figure S53. Top views of optimized geometries of **1**-**5** (*Z*)-isomers with the intramolecular H-bond distances shown in orange, calculated at the PCM-CAM-B3LYP/6-31G** level using acetonitrile as solvent.

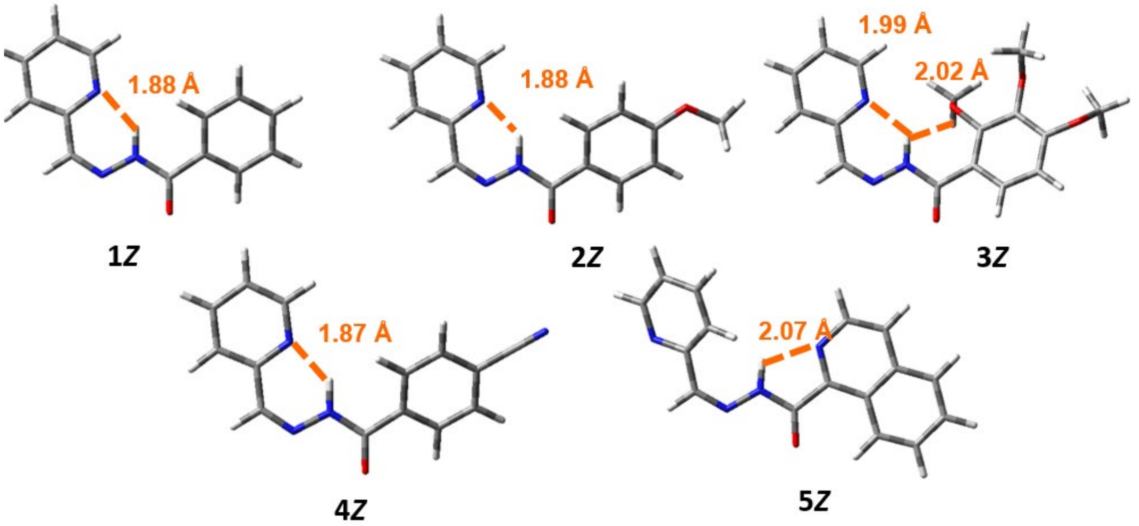

Figure S54. Top views of optimized geometries of **1**-**5** (*Z*)-isomers with the intramolecular H-bond distances shown in orange, calculated at the PCM-B3LYP/6-31G** level using acetonitrile as solvent.

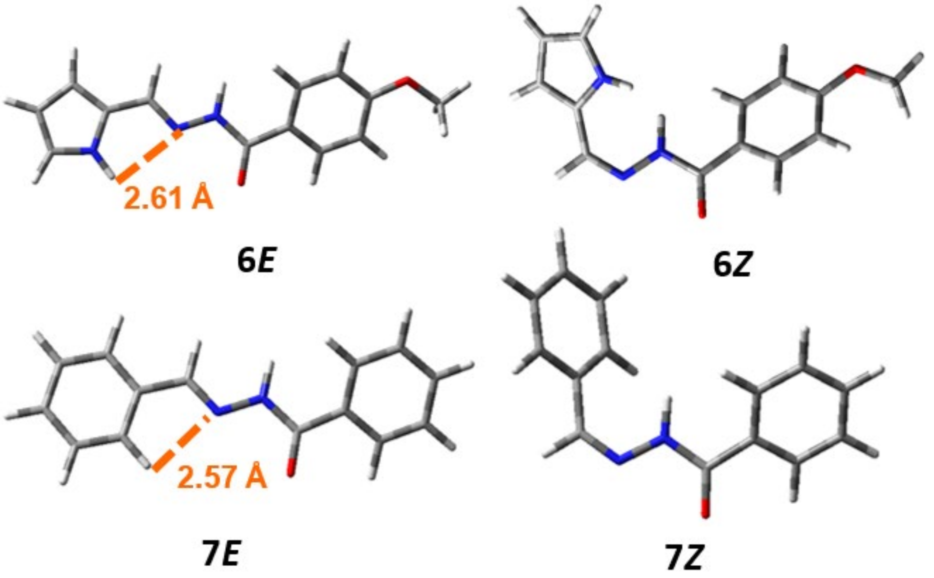

Figure S55. Top views of optimized geometries of **6** and **7** (*E*) and (Z)-isomers with the intramolecular H-bond distances shown in orange, calculated at the PCM-CAM-B3LYP/6-31G** level using acetonitrile as solvent.

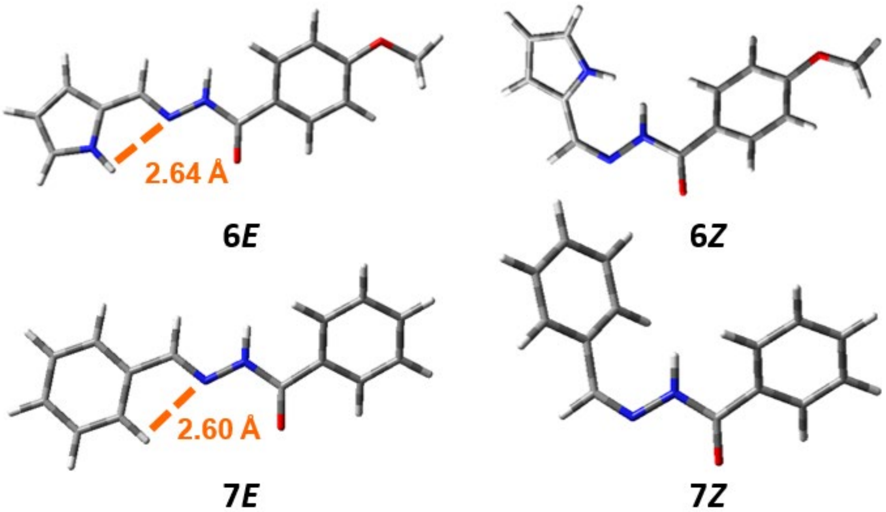

Figure S56. Top views of optimized geometries of **6** and **7** (*E*) and (Z)-isomers with the intramolecular H-bond distances shown in orange, calculated at the PCM-B3LYP/6-31G** level using acetonitrile as solvent.

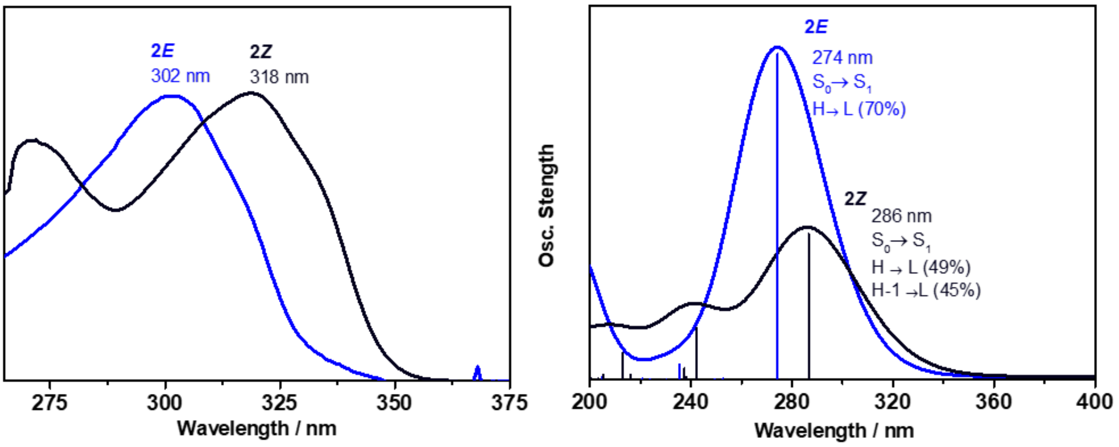

Figure S57. UV-Vis absorption spectra of the photoisomerization experiment of acylhydrazones **2** (left) and TD-DFT-calculated electronic absorption spectra for **2*E*** and **2*Z*** isomers, at the PCM-CAM-B3LYP/6-31G** level using acetonitrile as solvent (right).

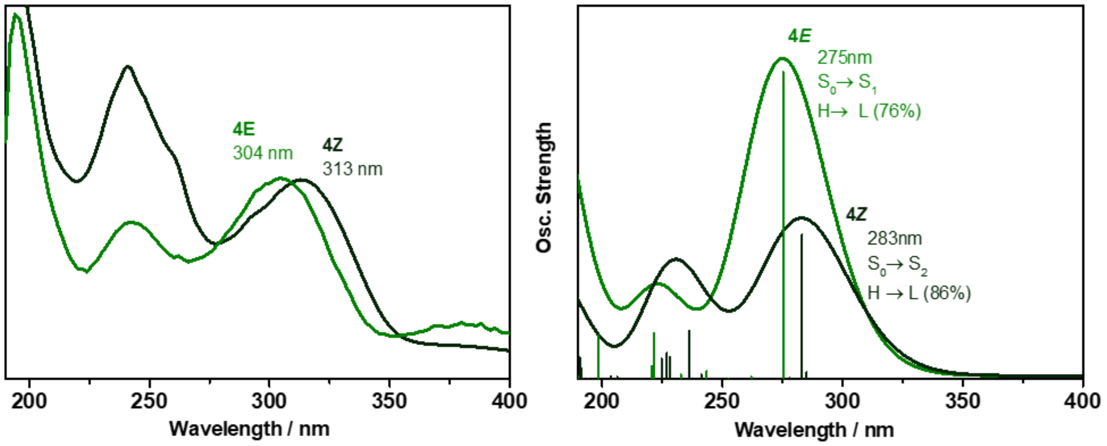

Figure S58. UV-Vis absorption spectra of the photoisomerization experiment of acylhydrazones **4** (left) and TD-DFT-calculated electronic absorption spectra for **4*E*** and **4*Z*** isomers, at the PCM-CAM-B3LYP/6-31G** level using acetonitrile as solvent (right).

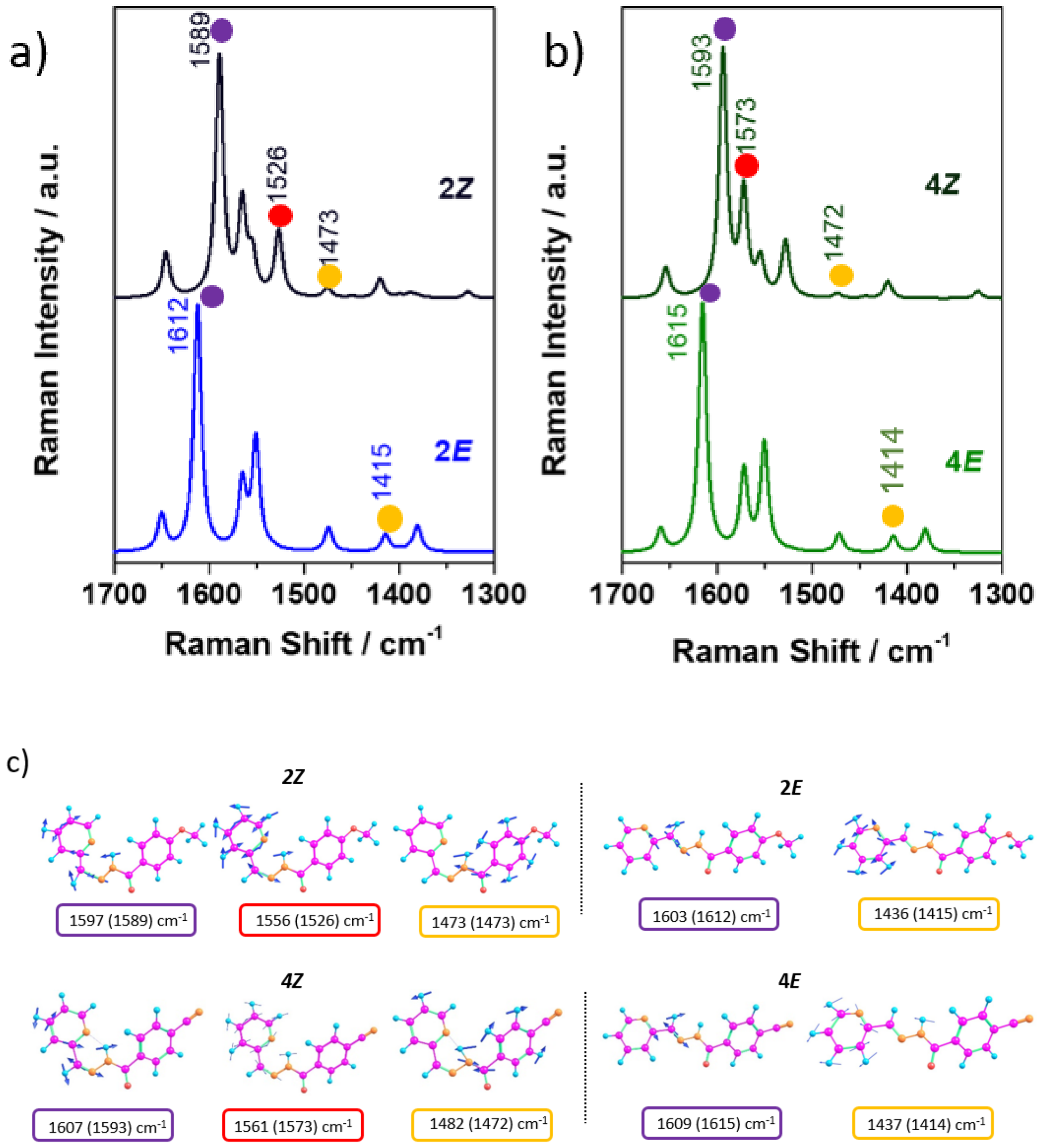

Figure S59. Theoretical Raman spectra for *E* and *Z* isomers of **2** (a) and **4** (b) compounds, calculated at PCM-CAM-B3LYP/6-31G** level using acetonitrile as solvent. Solid-state (c) Vibrational eigenvectors associated with the Raman features discussed in the text. The experimental and theoretical (in parentheses) wavenumbers are also shown.
